# Metformin pretreatment ameliorates busulfan-induced liver endothelial toxicity during haematopoietic stem cell transplantation

**DOI:** 10.1371/journal.pone.0293311

**Published:** 2023-10-26

**Authors:** Balaji Balakrishnan, Raveen Stephen Stallon Illangeswaran, Bharathi Murugan Rajamani, Arun Kumar Arunachalam, Aswin Anand Pai, Ezhilpavai Mohanan, Alok Srivastava, Vikram Mathews, Poonkuzhali Balasubramanian

**Affiliations:** 1 Department of Haematology, Christian Medical College, Vellore, India; 2 Centre for Stem Cell Research (CSCR), A Unit of InStem Bengaluru, Christian Medical College Campus, Vellore, India; University of Tennessee Health Science Center College of Medicine Memphis, UNITED STATES

## Abstract

The success of Haematopoietic cell transplantation (HCT) is often limited by regimen-related toxicity (RRT) caused by conditioning regimen drugs. Among different conditioning drugs, busulfan (Bu) and treosulfan (Treo), although widely used in HCT, exhibit different toxicity profiles, the mechanism of which is still unclear. Here we investigated the effects of Bu and Treo in endothelial cells. While both Bu and Treo induced DNA damage in endothelial cells, we observed Bu alone to induce oxidative stress and sustained activation of phospho-ERK1/2, leading to apoptosis. However, Treo-treated cells exhibited no oxidative stress/apoptosis of endothelial cells. Screening of pharmacological inhibitors of both ROS and p-ERK revealed that metformin effectively ameliorates Bu-mediated toxicity in endothelial cells. In Balb/c mice, we observed a significant reduction in bone marrow endothelial cells in Bu-treated mice compared to Treo-treated mice. Further, liver sinusoidal endothelial cells (LSEC) was damaged by Bu, which is implicated in liver vasculature and their functional capacity to uptake FITC-albumin. However, Treo-treated mice liver vasculature was morphologically and functionally normal. When mice were pretreated with metformin followed by Bu, LSECs damage was ameliorated morphologically and functionally. Bone marrow transplants done on these mice did not affect the engraftment of donor cells.

## Introduction

Hematopoietic cell transplantation (HCT) is an established curative treatment option for various hematological malignant and non-malignant diseases. However, the success of HCT is often limited by complications such as sinusoidal obstruction syndrome (SOS), graft versus host disease (GVHD), mucositis, transplant-associated microangiopathy (TAM), etc. [1, 2]. Although multifactorial, it is well recognized that a unifying factor in these complications is the off-target damage to endothelium caused by the direct action of conditioning regimen drugs (myeloablative/immunosuppressive) or total body irradiation (TBI) [3]. Hence they are grouped as regimen-related toxicity (RRT). These RRTs result in significant morbidity and mortality after Hematopoietic Cell Transplantation (HCT). The incidence and severity of RRTs are influenced by the intensity and type of conditioning regimen drugs [4–8].

Alkylating drugs remain a vital component of the conditioning regimen due to their myeloablative and immunosuppressive properties. The most frequently used alkylating agents are busulfan (Bu; 1,4-butanediol-dimethylsulfonate) and treosulfan (Treo; L-threitol-1,4-bis-methanesulfonate; dihydroxybusulfan). In combination with cyclophosphamide (Cy) or fludarabine (Flu), Bu has demonstrated efficient long-term donor engraftment in HCT patients, enabling better treatment outcomes. However, low exposure to Bu is associated with graft rejection and relapse, while high levels are associated with fatal SOS, neurologic and pulmonary toxicities [9–13], indicating a narrow therapeutic range.

Treosulfan-based conditioning regimens have shown a consistent pharmacokinetic profile with improved outcomes accompanied by a decrease in RRTs such as SOS in patients with myeloid malignancies, Myelodysplastic syndrome (MDS) [14–18], and bone marrow failure disorders [19]. Flu/Treo/thiotepa-based toxicity-reduced conditioning regimen for HCT in patients with high-risk β-thalassemia major has improved overall survival, engraftment rates and decreased the incidence of SOS when compared to Bu/Cy [20, 21] based regimen in our center. However, distinct gonadal [22] and skin toxicities [23] also appear to be associated with treo-based conditioning regimen. Despite these clinical observations, Bu or Treo remains the inevitable component of conditioning in patients undergoing HCT [14, 24, 25].

However, the exact mechanisms of Bu or Treo-mediated off-target toxicities remain unclear. Bu-based conditioning regimens have been shown to cause endothelial injury [26–28], liver inflammation through NLRP3 inflammasome activation [29], and increased circulating endothelial cells (CECs) in peripheral blood [27, 30, 31]. Since Bu is metabolized and eliminated via conjugation with glutathione by glutathione S-transferase alpha (GSTA1), particularly, Bu-mediated damage to endothelial cells has been attributed to the lack of GST enzymes [32, 33]. On the other hand, Treo has not been shown to cause endothelial or organ injury despite a significant Treo exposure found in the liver, lungs, kidney, or brain in a rat model [34].

Although Bu and Treo are structurally similar myeloablative agents, their endothelial toxicity profile differs significantly. The mechanism of endothelial toxicity profile differences between Bu and Treo remains unexplored. Here we assessed the molecular mechanisms behind the endothelial damage caused by Bu and Treo *in vitro* and *in vivo* to identify specific targets that can be modulated to alleviate damage to the endothelium without compromising the myeloablative potential of Bu.

## Results

### Differential cytotoxic effects of Bu and Treo on endothelial cells

**Fig 1:**
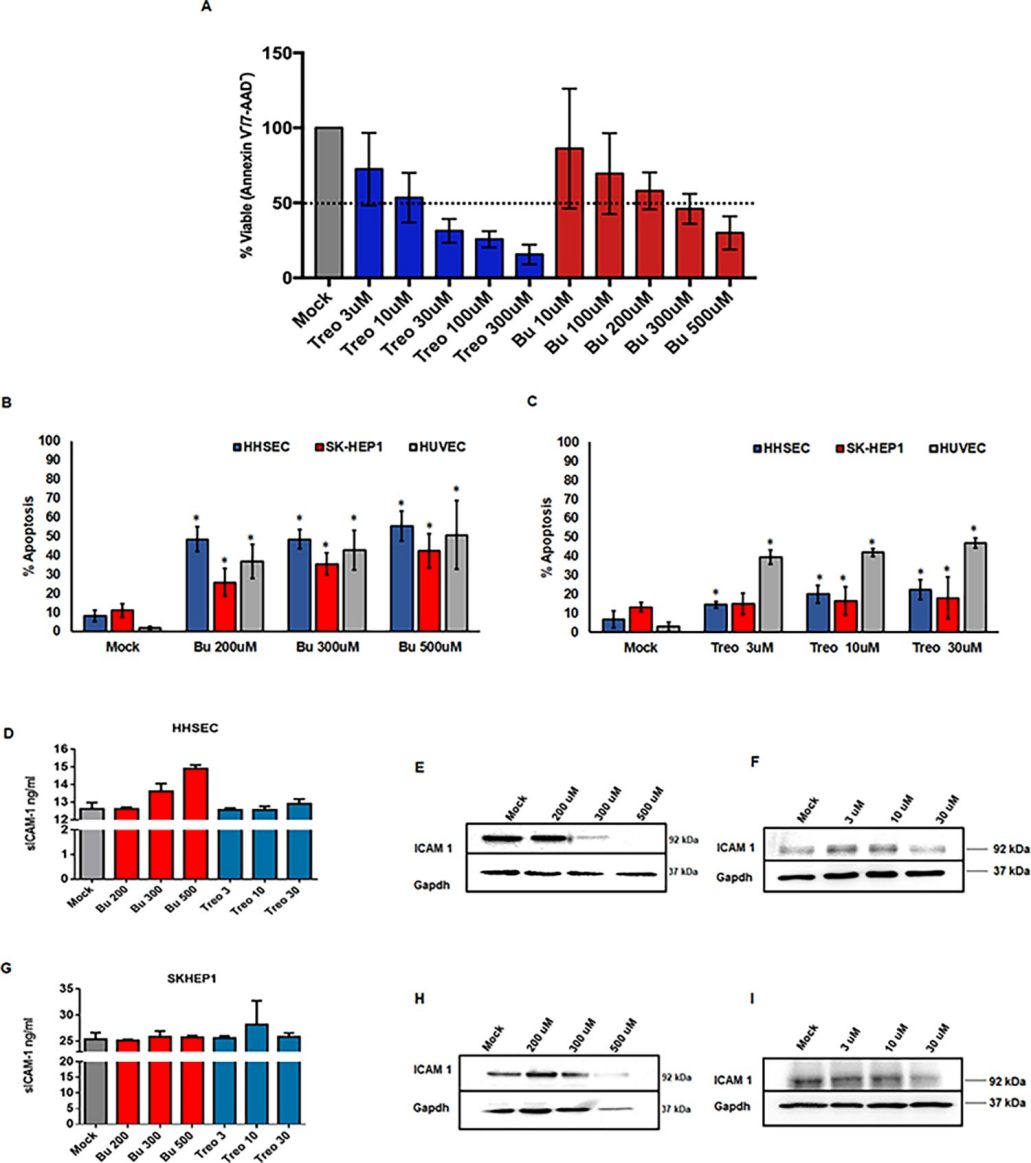
Bu and Treo induce apoptosis in endothelial cells of different origins. Peripheral blood mononuclear cells from healthy donors **(A)**, Primary human hepatic sinusoidal endothelial cells (HHSEC), and SK-HEP1, an immortalized cell line of hepatic endothelial origin and primary Human Umbilical Vein Endothelial Cells (HUVEC), were treated with Bu **(B)** or Treo **(C)** at different concentrations. Forty-eight hours post-treatment, the cells were harvested by trypsinization, stained with Annexin V-APC and 7-AAD, and analyzed by flow cytometry. Apoptotic cells were defined as Annexin V+/7AAD± and expressed as a percentage of the total cell number acquired. Data shown are mean± SD of three independent experiments in triplicates. The *P*-value was calculated by the Mann-Whitney *U* test. * p<0.05 vs. mock-treated cells Bu and Treo activate endothelial cells. Primary HHSECs and SK-HEP1 cells were treated with Bu or Treo at different concentrations. Forty-eight hours post-treatment, the cultures were evaluated for ICAM1 levels as a measure of endothelial cells activation. Culture supernatants from HHSECs and SK-HEP1 treated with Bu or Treo were evaluated for soluble ICAM1 (sICAM1) levels by ELISA. **(D)** and **(G).** Total protein lysates harvested from HHSECs treated with Bu or Treo were analyzed for ICAM1 levels by western blotting. **(E)** and **(F)**. Total protein lysates harvested from SK-HEP1 treated with Bu or Treo were analyzed for ICAM1 levels by western blotting **(H)** and **(I)**.

The concentrations of two different drugs, Bu and Treo, were chosen based on previous reports demonstrating a decrease in the viability of leukemic cells by 70–90% [35]. Based on these reports, a range of varying concentrations of Bu and Treo were initially evaluated on peripheral blood mononuclear cells (PBMNCs) from healthy donors. We observed >50% cytotoxicity in PBMNCs treated with Treo at 30uM and Bu at 300uM for 48h (Fig 1A). The concentrations at which Bu and Treo are cytotoxic to hematopoietic cells *in vitro* were further used to investigate their off-target toxicity on endothelial cells (i.e.) primary HHSECs, HUVEC, and SK-HEP1. We evaluated the endothelial cells by treating them with Bu (200, 300 & 500μM) or Treo (3, 10 & 30μM). Forty-eight hours post-Bu treatment, we observed a significant dose-dependent increase in apoptosis in all three endothelial cells (with a maximum of up to 55% in HHSEC, up to 43% in SK-HEP1 cells, and up to 51% in HUVEC) (Fig 1B). Treo treatment, even at the highest concentration (30uM), induced minimal apoptosis in liver-specific endothelial cells (22% in HHSEC and 18% in SK-HEP1) (Fig 1C). While Treo at 30uM reduced the viability of primary CD34+cells by 63%, even higher concentrations are required to reduce the viability of liver-specific endothelial cells (S1A and S1B Fig). Interestingly, we observed dose-dependent apoptosis (up to 47%) in HUVEC cells treated with Treo (Fig 1C). These results indicate that the conditioning regimen drugs Bu and Treo are differentially cytotoxic to the endothelial cells, and Treo, in particular, is differentially cytotoxic to different endothelial cell types.

**Fig 2:**
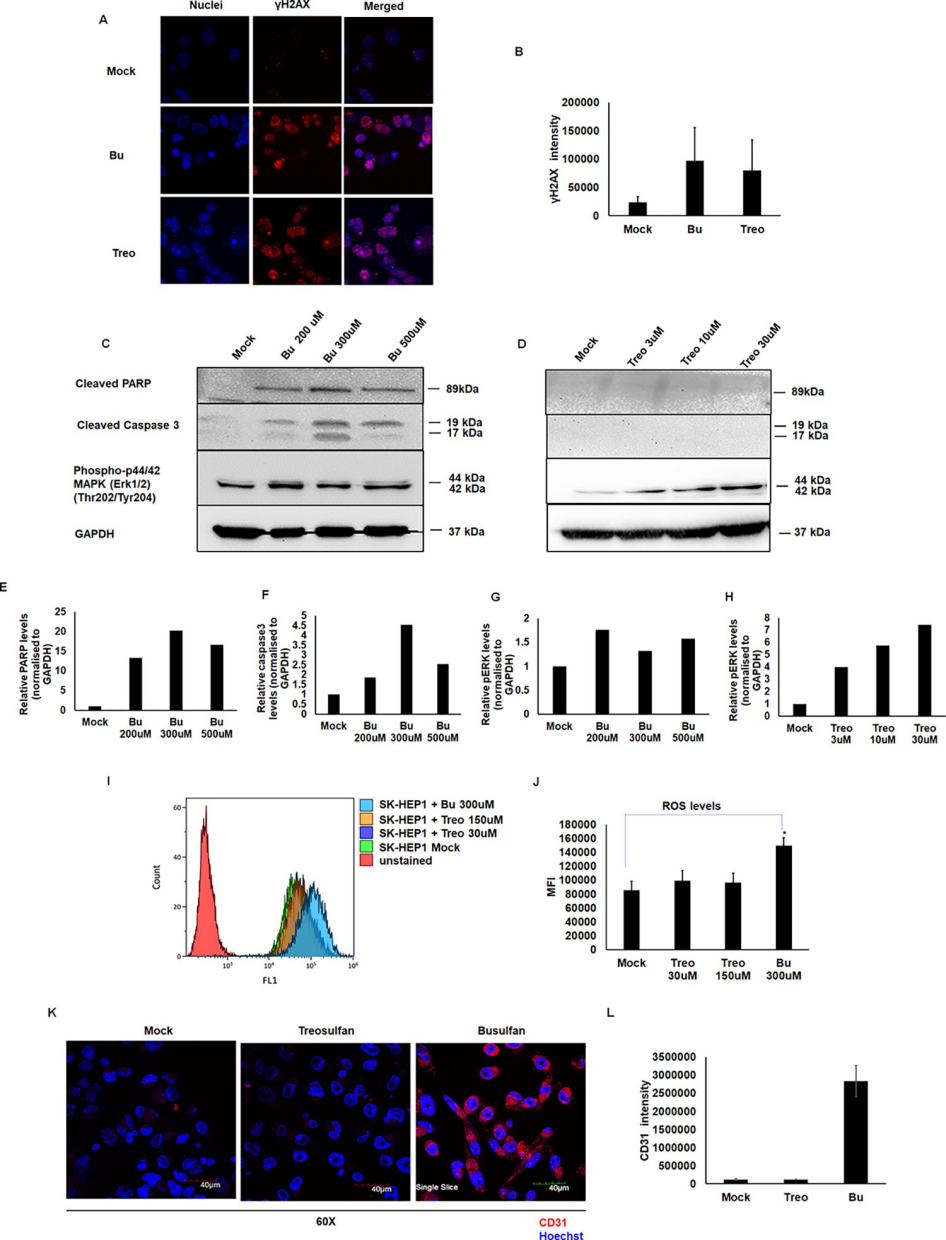
Bu, but not Treo, induces ROS-mediated apoptosis. SK-HEP1 cells treated with Bu or Treo were probed for γ-H2AX (pink) as a measure of DNA damage. **A.** Immunofluorescence images of SK-HEP1 cells treated with Bu or Treo for 6 hours and probed for γ-H2AX (pink) and their nuclei counterstained with hoechst33342. **B.** Levels of γ-H2AX quantified using ImageJ. SK-HEP1 was treated with Bu or Treo at different concentrations. The protein lysates were harvested at twelve hours post-treatment for analysis of the protein levels of cleaved PARP, cleaved caspase 3, p44/42 MAPK(Erk1/2), and gapdh post-Bu **(C)** and Treo treatment **(D).** The band intensities of all test and control conditions were calculated by densitometric analysis using Image J software **(E, F, G, and H)**. Reactive oxygen species (ROS) levels were assessed by measuring DCFDA fluorescence by flow cytometry. **I**. Representative histogram of ROS levels post-drug treatment. **J**. Graph showing mean fluorescence intensity (MFI) of DCFDA indicating ROS levels post-Bu/Treo treatment in SK-HEP1 cells. **Bu induces capillarization of liver endothelial cells.** SK-HEP1 cells are negative for CD31 expression. The cells were treated with Bu or Treo for twelve hours. The cells were then fixed in 4% paraformaldehyde, blocked with 1% BSA, and probed with CD31 antibody. **K.** Immunofluorescence images of SK-HEP1 cells treated with Bu or Treo and were probed with CD31 antibody. **L.** CD31 expression levels quantified by ImageJ. Data shown are mean± SD of three independent experiments in triplicates. The *P*-value was calculated by the Mann-Whitney *U* test. * p<0.05 vs. mock-treated cells.

Further, as a marker for endothelial cell activation, we evaluated ICAM1. In liver endothelial cells treated with Bu, we observed an increase in soluble ICAM1 expression in culture supernatants (Fig 1D and 1G) and a concomitant dose-dependent decrease in total cellular ICAM1 expression (Fig 1E and 1H). While soluble ICAM1 expression did not change with Treo treatment, total cellular ICAM1 decreased at the highest concentration (30uM) (Fig 1F and 1I). However, we observed almost similar levels of phospho-γH2AX post-Bu or Treo treatment, indicating their alkylating activity (Fig 2A and 2B). These results demonstrate that Bu and Treo alkylate DNA, induce DNA damage but differentially exert cytotoxicity on liver-specific endothelial cells.

### Bu induces ROS mediated apoptosis and capillarization of liver endothelial cells

Next, we examined the apoptotic effects of Bu or Treo on SK-HEP1 cells. We observed an increased cleaved PARP1 and cleaved caspase-3 in Bu-treated cells but not in Treo-treated cells. However, phosphorylated p44/42-MAPK (Erk1/2) expression was observed in both Bu and Treo-treated cells (Fig 2C–2H). It is well known that ERK1/2 activation leads to cell survival, growth, and differentiation. It is also known that sustained activation of ERK1/2 by oxidative stress promotes cell death. Indeed, we observed significantly increased reactive oxygen species (ROS) levels in Bu-treated cells but not in Treo-treated cells, even at the highest concentration (Fig 2I and 2J). However, the mitochondrial membrane potential was only modestly altered, which was statistically insignificant (S5 Fig). These results demonstrate that Bu induced ROS and sustained ERK1/2 activation, which leads to apoptosis executed by cleaved PARP1 and caspase 3. We also observed a phenotypic change in Bu-treated SK-HEP1 cells. The untreated cells are negative for CD31 expression (S2 Fig). However, we observed a prominent CD31 surface expression upon Bu treatment but not in Treo treatment (Fig 2K and 2L), indicating that the liver endothelial cells undergo capillarization, a process where the cells dedifferentiate because of damage.

### Pharmacological inhibition of ROS and phospho-ERK (1/2) ameliorates Bu-induced damage to endothelial cells

**Fig 3:**
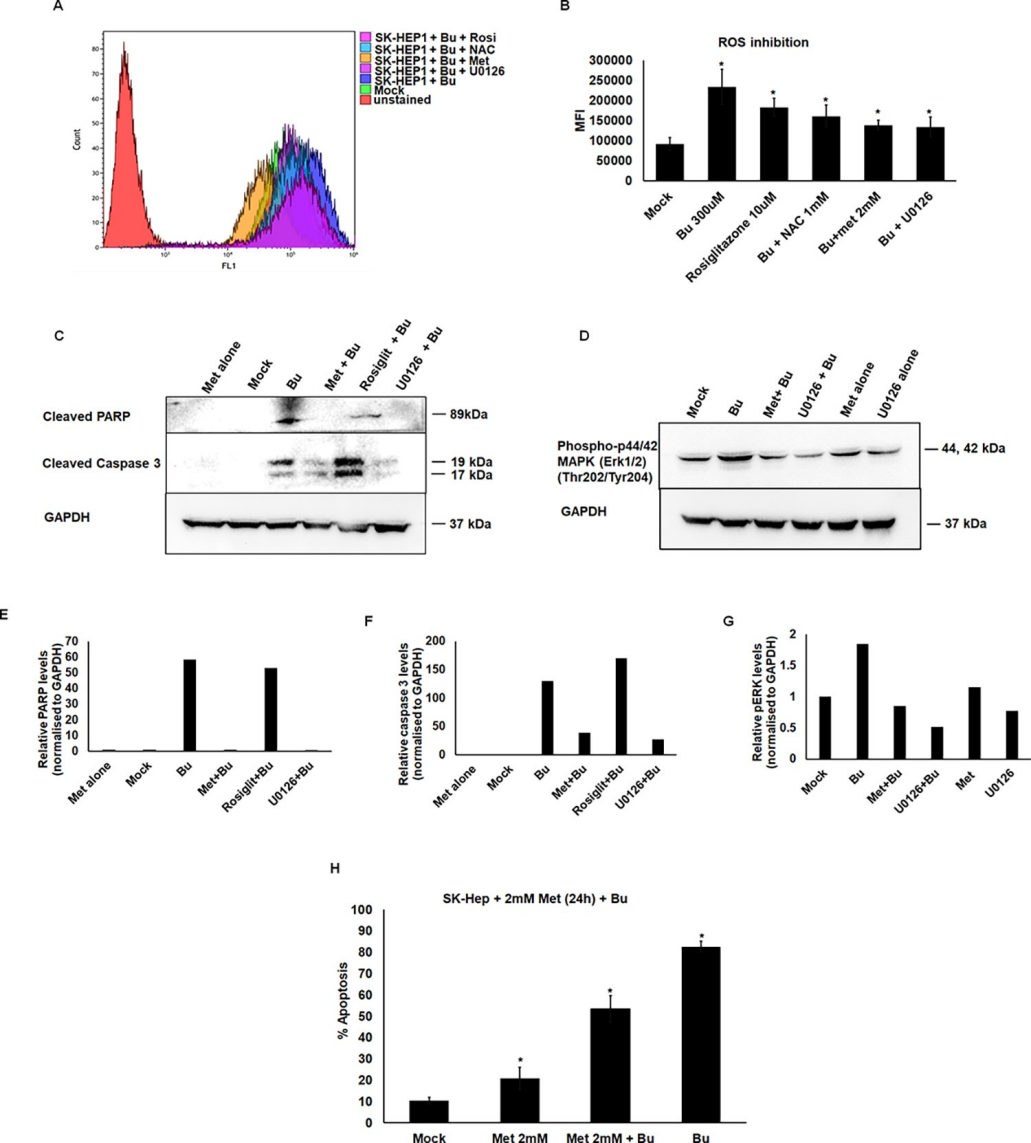
Pharmacological inhibition of ROS and phospho-Erk(1/2) ameliorates Bu-induced endothelial cell damage. SK-HEP1 cells were pretreated with N-acetyl cysteine (NAC) (1mM) or rosiglitazone (1mM) or metformin (2mM) or U0126 (10uM). One hour post-treatment, the cells were incubated with Bu (300uM). Eight hours post-treatment, the cells were analyzed for ROS levels by measuring DCFDA fluorescence by flow cytometry. **A.** representative histogram overlay of inhibition of ROS assay. **B.** Graph showing MFI levels indicating ROS levels post-drug pretreatment. **C** and **D.** Western blot images showing levels of cleaved PARP, cleaved caspase 3, phospho-Erk(1/2) post inhibitors pretreatment. **E, F, and G.** Graphs showing relative levels of the proteins normalized to GAPDH by densitometric analysis using ImageJ software. **H.** Apoptosis percentages of SK-HEP1 cells pretreated with metformin (2mM) for 24 hours and Bu for 48h. Data shown are mean± SD of three independent experiments in triplicates. The *P*-value was calculated by the Mann-Whitney *U* test. * p<0.05 vs. mock-treated cells.

Based on our findings that Bu induces apoptosis through DNA damage, sustained activation of ROS, p-ERK (1/2), and terminal execution through PARP1/caspase3, we next screened for pharmacological inhibitors (N-acetyl cysteine (NAC), rosiglitazone, metformin, and U0126) of both ROS and p-ERK. Among them, we observed pretreatment of SK-HEP1 cells with metformin or U0126 significantly decreased cellular ROS levels compared to Bu treatment alone (Fig 3A and 3B). We also observed reduced levels of cleaved PARP1, cleaved caspase3, and pERK (1/2) upon metformin and U0126 pretreatments (Fig 3C–3G). Since sustained inhibition of pERK (1/2) was observed in the U0126 treatment alone (Fig 3D), we evaluated metformin in further experiments. However, metformin pretreatment for 24h prior to Bu treatment significantly decreased total apoptosis percentage (53%) compared to Bu alone (82%) (Fig 3H).

### Myeloablative doses of Bu and Treo are differentially toxic to endothelial cells of bone marrow and liver *in vivo*

**Fig 4:**
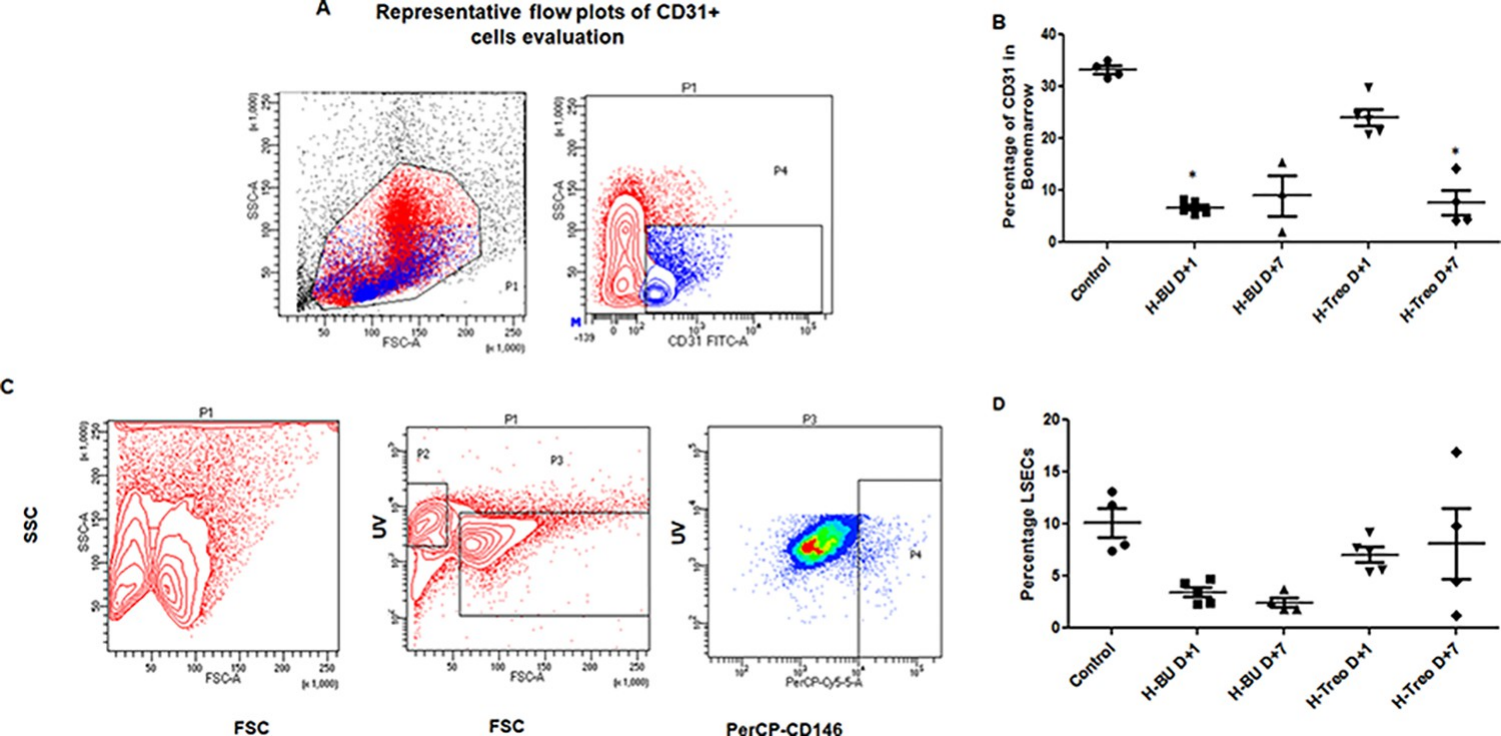
Myeloablative doses of Bu and Treo are differentially toxic to different endothelial cells *in vivo*. Groups of Balb/c mice (n = 5) were injected with Bu (25mg/kg/day) for 4 days or Treo (1000mg/kg/day) for 3 days. On day+1 and day+7 post-drug treatment, the mice were euthanized and analyzed for myeloablation and endothelial damage. Untreated mice were used as controls. **A and B.** Evaluation of CD31+ bone marrow endothelial cells from treated or untreated mice by flow cytometry. **C.** Gating strategy to evaluate CD146+ liver sinusoidal endothelial cells from livers of treated or untreated mice. **D**. Percentage CD146+ liver sinusoidal cells from livers of treated or untreated mice. The *P*-value was calculated by the Mann-Whitney *U* test. * p<0.05 vs. untreated mice.

We next evaluated Bu and Treo *in vivo* in Balb/c mice at previously reported myeloablative concentrations. We observed a profound decrease in bone marrow cellularity in Bu or Treo-treated mice (S3 Fig). We wished to evaluate the extent of bone marrow endothelial damage post-myeloablation without HCT support. Mice treated with Bu showed a decrease in BMECs on day+1 [median 6.2 (range; 5.4–7.9%); *p = 0*.*0066*] and on day+7 [median 9.4 (range; 2.0–15.4%) *p = 0*.*05*]. However, mice treated with Treo slowly decreased BMECs from day+1 [median 23.6 (range; 20.9–29.8%); *p = 0*.*9*] to day+7 [median 6.1 (range; 4.2–14.2%) *p = 0*.*0069*] when compared to untreated control [median 33.15 (range; 31.7–35%)]. These results (Fig 4A and 4B) indicate that despite their intended marrow toxicity, both Bu and Treo exhibit off-target endothelial toxicity, although differentially.

We then evaluated the percentage of liver sinusoidal endothelial cells (LSECs) by flow cytometry (Fig 4C and 4D). Percentage of LSECs were decreased by Bu treatment on day+1 [median 3.6 (range; 2.3–4.7%); *p = 0*.*048*] and day+7 [median 2.15 (range; 1.8–3.7%); *p = 0*.*01*] while Treo treatment resulted in relatively less damage on both day+1 [median 7.4 (range; 5.4–9.2%); *p = 0*.*9*] and day+7 [median 7.15 (range; 1.2–16.8%); *p = 0*.*9*] when compared to untreated control mice [median 9.9 (range; 7.4–13.1%)] (Fig 4D). These results corroborate our *in vitro* findings that Bu and treo are differentially toxic to different endothelial cells.

### Bu induces capillarization of liver sinusoidal endothelial cells and damage to the spleen

**Fig 5:**
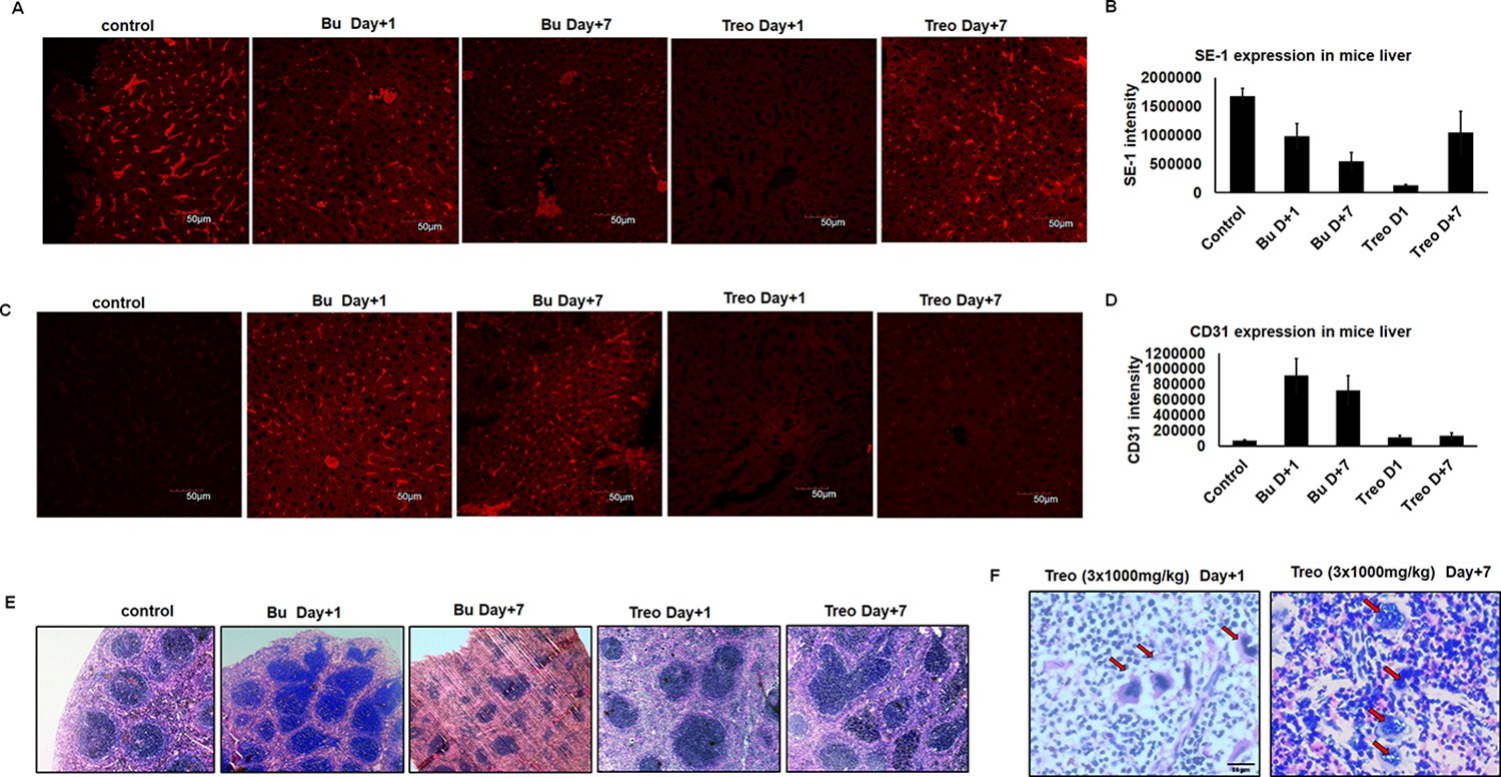
Treo does not capillarize liver sinusoidal endothelial cells but induces extramedullary hematopoiesis. Groups of Balb/c mice (n = 5) were injected with Bu (25mg/kg/day) for 4 days or Treo (1000mg/kg/day) for 3 days. On day+1 and day+7 post-drug treatment, the mice were euthanized and analyzed for myeloablation and endothelial damage. Untreated mice were used as controls. **A.** Immunofluorescence images of treated or untreated mice livers probed for LSECs using SE-1 antibody (red). **B.** SE-1 levels in treated or untreated mice. **C.** Immunofluorescence images of treated or untreated mice livers probed for CD31 (red). **D.** CD31 levels in treated or untreated mice. **E.** Hematoxylin and Eosin (H&E) staining of spleen sections from treated or untreated mice. **F.** Zoomed in H&E images of Treo-treated mice spleen sections. Red arrows indicate extramedullary hematopoiesis.

The damage to LSECs was further assessed using the SE-1 antibody, which detects LSECS specifically. Immunofluorescence analysis of LSECs using SE-1 antibody (that specifically labels SE-1 antigen of LSECs) revealed that the LSECs are highly damaged upon Bu treatment and relatively less damaged upon Treo treatment (Fig 5A and 5B). It is known that normal LSECs do not express CD31 on their surface. However, upon capillarization, differentiated LSECs undergo dedifferentiation with increased CD31 expression, loss of fenestration, and altered endothelial cell permeability. We observed CD31 expression in liver sections of Bu-treated mice on both day+1 and day+7. However, Treo treatment did not result in CD31 expression (Fig 5C and 5D). These results indicate that Bu induces capillarization of LSECs but not Treo.

Next, we wished to evaluate the mice spleen upon Bu and Treo treatment. Hematoxylin & eosin (H&E) staining of spleen sections revealed white pulp atrophy in Treo-treated mice at day+1, while Bu-treated mice showed profound atrophy only at day+7. However, we observed increased cellularity of splenic white pulp in Treo-treated mice on day+7, indicating possible recovery (Fig 5E). Interestingly, we also observed megakaryocytes in Treo-treated mice, indicating megakaryopoiesis that contributes to extramedullary hematopoiesis in the spleen (Fig 5F). However, this phenomenon was absent in Bu-treated mice. These results suggest a probable recovery mechanism after Treo treatment.

### Metformin pretreatment ameliorates Bu-induced damage to the liver and spleen

**Fig 6:**
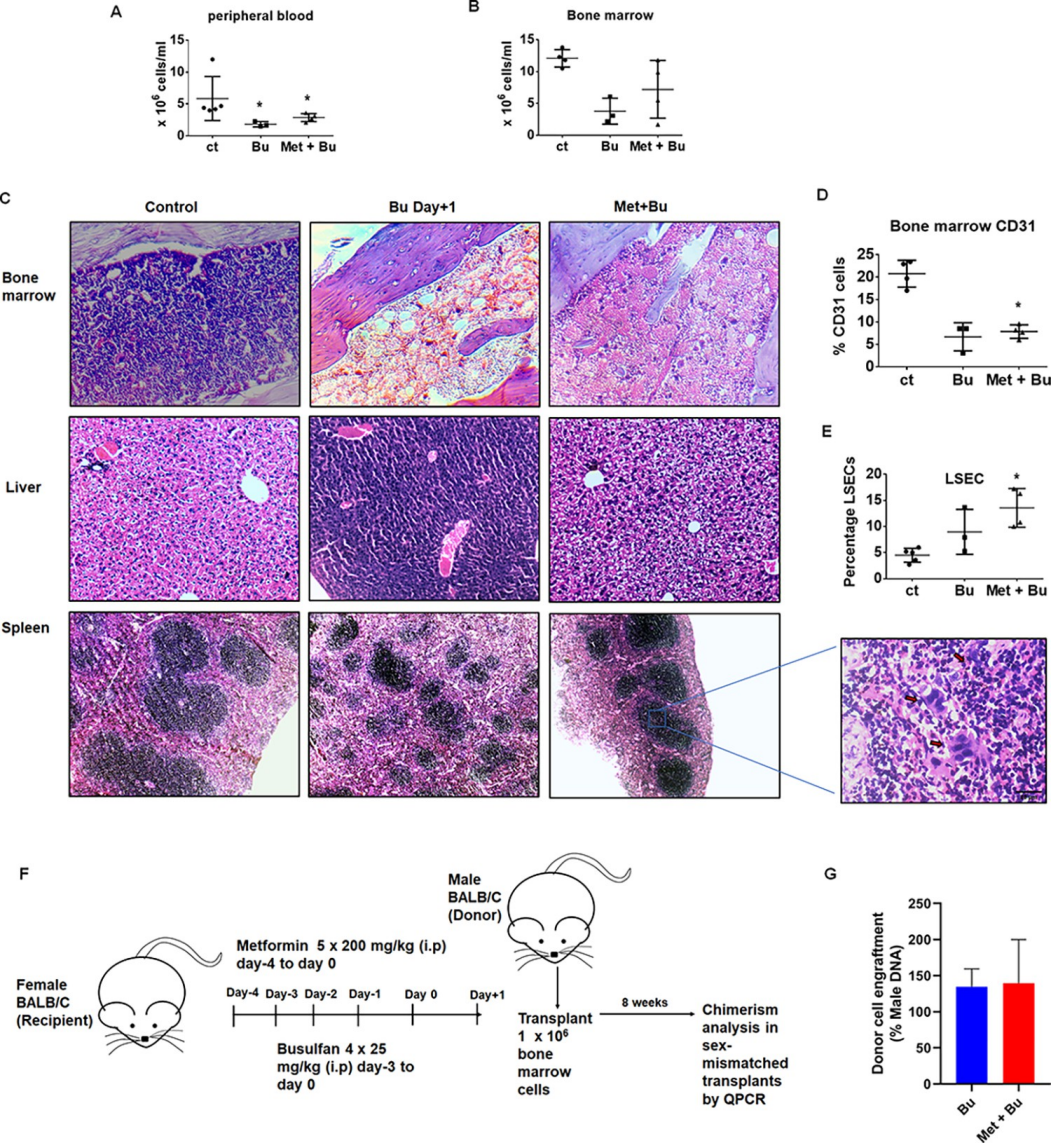
Metformin pretreatment ameliorates Bu-induced damage to the liver and spleen. Groups of Balb/c mice (n = 5) were injected with Bu alone (25mg/kg/day) for 4 days or pre-treated with metformin (200 mg/kg) on day-4 and days -3, -2, -1, 0 one hour prior to Bu treatment (metformin+Bu group). On day+1 post-drug treatment, the mice were euthanized and analyzed for myeloablation and endothelial damage. Untreated mice were used as controls. **C.** H&E stained bone, liver, and spleen sections from treated or untreated mice. Zoomed-in image of H&E stained spleen section from metformin+Bu treated mice showing megakaryocytes. **D.** Percentage CD31+ endothelial cells from the bone marrow of treated or untreated mice. **E.** Percentage LSECs from livers of treated or untreated mice. **Metformin pretreatment does not affect engraftment post-transplantation. F.** Schematic of the transplantation procedures done in Balb/c mice either treated alone or with metformin and Bu. G. Donor cell engraftment percentage in bu treated or metformin+Bu treated mice at 8 weeks post-transplantation. The P-value was calculated by the Mann-Whitney *U* test. * p<0.05 vs. untreated mice.

Based on our *in vitro* results that metformin pretreatment protects Bu-induced damage, we wished to evaluate the same in the Balb/c mice model. Metformin pretreatment did not affect the myeloablative effect of Bu. This was implicated in bone marrow WBCs [Bu alone: median 3.1 (range; 2.2–6.1 x 10^6^ cells/ml); metformin+Bu: median 7.7 (range; 1.7–11.8 x 10^6^ cells/ml)] (Fig 6B and 6C) and peripheral blood counts [Bu alone: median 1.7 (range 1.5–2.3 x 10^6^ cells/ml); metformin+Bu: median 2.9 (range 2.1–3.6 x 10^6^ cells/ml)] (Fig 6A). However, the percentage of BMECs improved in the metformin pretreatment group (median 7.9 (range; 6–9.6%) when compared to Bu alone (median 8.5 (range; 3.1–8.5%) (Fig 6D). Moreover, Bu or Treo-mediated increase in the expression of endothelial cells activation markers (VCAM1, ICAM1, VWF, and TNFR1) were significantly downregulated upon metformin pretreatment (S4 Fig). H&E staining of liver sections of metformin-pretreated mice showed normal hepatocyte architecture with cords of hepatocytes and eosinophilic cytoplasm. In contrast, the Bu-treated mice showed perivascular hepatocytes with effaced architecture and granular cytoplasm (Fig 6C). Similarly, percentage LSECs in the metformin pretreatment group were improved [median 13.5 range (10–17.2%)] compared to Bu alone [median 7.9 range (5.3–13.7%)] (Fig 6E).

While we observed decreased splenic cellularity in red and white pulps of Bu-treated mice, metformin pretreatment improved splenic cellularity (Fig 6C). Surprisingly, we also observed megakaryocytes in splenic sections of metformin-pretreated mice, indicating probable extramedullary hematopoiesis.

Transplantations in mice treated with Bu alone or metformin plus Bu resulted in almost similar engraftment potential, as evidenced by long-term engraftment analysis (Fig 6F and 6G). Collectively, these results indicate that metformin pretreatment improves Bu-mediated damage to bone marrow, liver, and spleen while still maintaining the myeloablative effects of Bu.

### Metformin pretreatment improves liver vascular functionality and prevents LSEC capillarization

**Fig 7:**
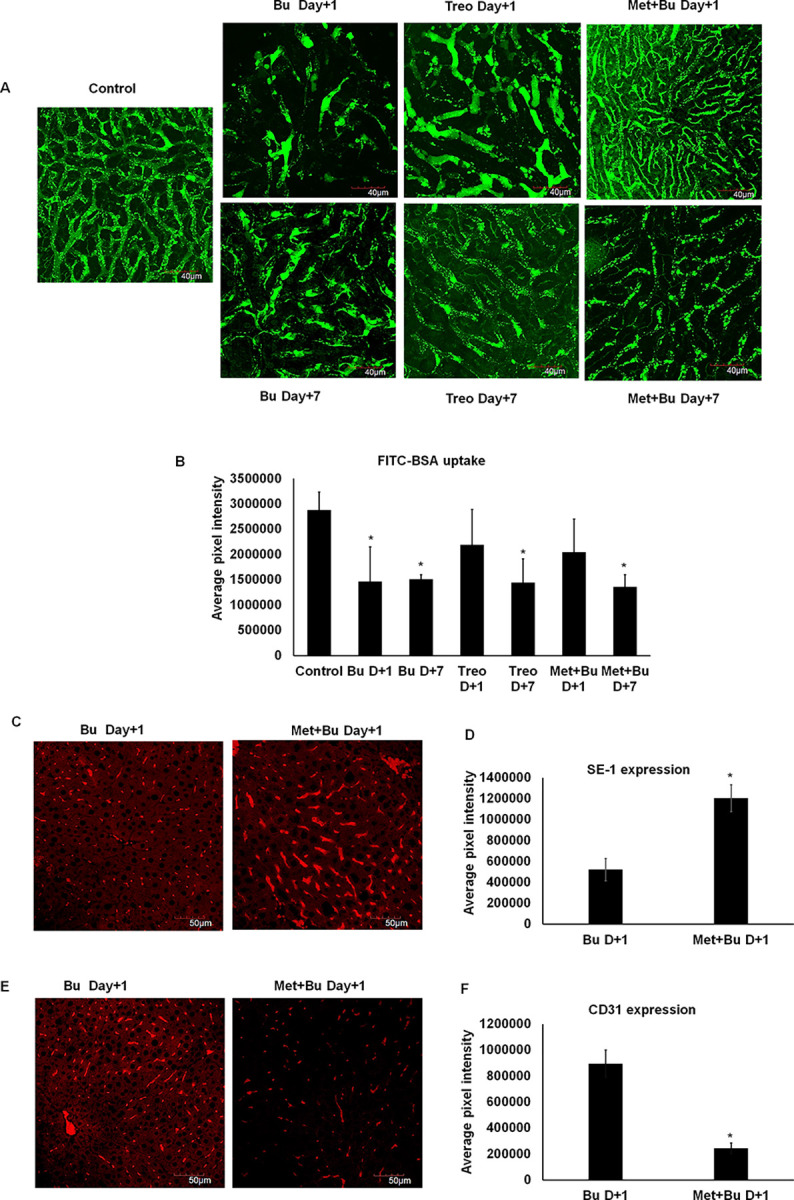
Metformin pretreatment improves liver vascular functionality and prevents LSEC capillarization. **A.** Groups of Balb/c mice (n = 5) treated with Bu, Treo, or metformin+Bu were administered FITC-albumin (1mg/mouse) intravenously at indicated time points. Thirty minutes post-administration, the mice were euthanized, liver lobes harvested, and whole mounts of liver lobes were imaged using Olympus FV1000 laser scanning confocal microscope at 60x oil immersion objective. **A.** Confocal z-stacked images showing FITC-albumin uptake by liver vasculature. **B.** Quantification of FITC-albumin uptake by liver vasculature using ImageJ. * p<0.05 vs untreated mice. **C** and **D.** Immunofluorescence images showing LSECs probed using SE-1 antibody post bu or met+Bu treatments and their levels quantified by ImageJ. **E** and **F.** Immunofluorescence images showing LSECs probed using CD31 antibody post bu or met+Bu treatments and their levels quantified by ImageJ. The *P*-value was calculated by the Mann-Whitney *U* test. * p<0.05 vs. Bu-treated mice.

Next, we wished to determine liver vasculature functionality by the ability of liver endothelial cells to uptake FITC-conjugated albumin. Compared to untreated mice, Bu-treated mice on day+1 and day+7 showed disrupted patterns of FITC+ area, indicating a profound decrease in vascular FITC albumin uptake. Upon Treo treatment, on day+1, we observed dilation of liver vasculature as shown by increased FITC+ area, while on day +7, this alteration in vessel morphology was reduced. Metformin pretreatment prior to Bu improved vascular morphology with continuous FITC uptake, and no dilations were observed (Fig 7A and 7B). While we earlier showed that Bu severely damaged LSECs and induced capillarization, here we observed that metformin pretreatment prior to Bu prevents damage to LSECs as shown by SE-1 expression (Fig 7C and 7D) and also prevents capillarization of LSECs as demonstrated by CD31 expression (Fig 7E and 7F). These results indicate that metformin pretreatment improves liver vasculature morphology and functionality and prevents LSEC capillarization. Capillarization is a process of dedifferentiation of LSECs characterized by the loss of fenestration and changes in surface markers expression, which occurs as an early event during a liver injury in animal models and patients [36–39].

## Discussion

Although alkylating agents are used in conditioning regimens due to their stem cell destructive myeloablative potential, extramedullary toxicity is still a concern. Bu, the most frequently used alkylating agent, and Treo, a relatively newer agent, appear almost similar structurally. Bu-induced bifunctional DNA alkylation involves intra/inter strands reacting with guanines (N7) through nucleophilic substitution reaction (SN2) and DNA-protein linkages leading to profound cytotoxicity [40]. Although Treo has an identical chain length as that of Bu, exhibits a different alkylating mechanism that is still unclear. It is known that Treo chemically transforms into epoxy derivatives {(S, S)-DEB and S, S-(EBDM)} which function as mono and bifunctional alkylating agents in inducing cytotoxicity [41].

The alkylating effects of Bu and Treo remain so profound in most hematopoietic cells, including primitive and committed stem cells, that they remain as important conditioning regimen drugs. However, in terms of extramedullary toxicities, the Bu-based regimen is more often associated with RRTs such as sinusoidal obstruction syndrome (SOS), severe neurologic and pulmonary toxicity compared to the Treo-based regimen [35]. One of the ways in which such early post-transplant complications occur is the initial damage to the endothelium of different organs caused by the conditioning regimen itself. Nevertheless, these drugs are still being used in HCT for various conditions. The reason behind this differential extramedullary toxicity profile of these two structurally similar drugs remains unexplored. Here we compared Bu and Treo to better understand the molecular mechanisms of their liver toxicity profile *in vitro* and *in vivo*.

When the cytotoxic effects of Bu and Treo were evaluated on three endothelial cell types (i.e.) primary HHSECs, HUVEC, and SK-HEP1, an immortal cell line of hepatic endothelial origin, we observed Bu-induced increased apoptosis in liver-specific endothelial cells when compared to Treo. However, in HUVEC, both Bu and Treo induced prominent apoptosis, demonstrating that the cytotoxic effects of these drugs are endothelial cell type-specific. Although the *in vitro* concentrations of these two drugs are not equimolar, it should be noted that they are cytotoxic to CD34+ cells, PBMNCs, and leukemic cells at these concentrations. This explains that the differential cytotoxicity of Bu and Treo observed in liver-specific endothelial cells closely mimics the off-target toxicity of these drugs observed *in vivo*. Our results corroborate this finding in that both Bu and Treo induce DNA damage, as seen by phospho-γH2AX expression, whereas Bu activates endothelial cells by inducing ICAM1 expression in endothelial cells. Apart from DNA crosslinking, it is also well recognized that Bu induces oxidative stress. However, few reports of Bu-mediated apoptosis through molecular mechanisms involving oxidative stress exist. Here we show that Bu induces sustained ROS production and increased activation of p-ERK (1/2), leading to apoptosis executed via cleaved PARP1 and caspase3. Although such ROS production was not observed with Treo, we did observe dose-dependent p-ERK (1/2) activation. Earlier studies suggested that activation of ERK (1/2) could promote cell survival, growth, and differentiation [42].

In contrast, oxidative stress-induced sustained activation of p-ERK(1/2) could promote cell death [43, 44], demonstrating that ERK (1/2) activation is a double-edged sword. Thus, our results suggest that Treo-mediated ERK (1/2) activation has a protective effect, while Bu-mediated sustained activation of ROS and ERK(1/2) leads to apoptosis. Pharmacological inhibition of ROS and p-ERK (1/2) activation by metformin or U0126 ameliorated Bu-mediated toxicity to liver endothelial cells. To our knowledge, this is the first study showing metformin pretreatment could ameliorate Bu-mediated LSEC toxicity. Based on our results, we believe that metformin could be repurposed and used in the HSCT setting to minimize liver toxicity without compromising engraftment potential.

Interestingly, upon Bu treatment, we also observed capillarization of liver-specific endothelial cells *in vitro* and *in vivo*, as observed by increased surface CD31 expression, a marker for capillarization of LSECs [45]. Capillarized LSECs undergo dedifferentiation characterized by defenestration and alterations in phenotype and functions. Apart from the phenotypic CD31 expression change, we also observed these cells to have decreased ability to take up macromolecules like albumin, indicating affected endocytosis function of liver endothelial cells. Generally, CD31 signaling on vascular endothelium has been reported to confer protection from extrinsic (TNFα, CTL induced) [46] or intrinsic apoptosis (toxic drug exposure) [47, 48] through Erk/Akt activation. In contrast, our results indicate that in LSECs, upon Bu treatment, capillarize, increase surface CD31 expression, and undergo apoptosis through sustained activation of oxidative stress and p-ERK (1/2).

Administration of Bu or Treo in Balb/c mice had the intended myeloablative effect until day+7 without any rescue by transplantation. However, differential damage to bone marrow and liver endothelial cells was also observed as off-target toxicity. Changes in percentages and morphology of LSECs were more prominent with Bu than with Treo. More importantly, capillarization of LSECs in Bu-treated mice but not in Treo-treated mice recapitulates our *in vitro* findings that Bu capillarize and promotes apoptosis of LSECs. Notably, the Bu-based conditioning regimen in HCT is more often associated with hepatic SOS than the Treo-based regimen [6, 9, 17, 49, 50]. However, we did not observe any of the clinical characteristics of SOS, such as hepatomegaly, jaundice, weight gain, and ascites, in our mice model of the conditioning regimen. This could be because of multiple factors like combinations of conditioning regimen drugs, transplantation, and liver status due to underlying disease conditions. Nevertheless, we did observe injury to LSECs, the preceding event in the SOS pathophysiology involving damage to LSECs [51]. Pharmacological inhibition of Bu-mediated ROS and pERK(1/2) using metformin ameliorated injury to LSECs as observed by improvements in morphology, phenotype, and functional endocytic abilities.

Metformin is a biguanide widely used as an antidiabetic drug [52]. Metformin has also been demonstrated to exert anti-inflammatory [53, 54] and anti-oxidant [55, 56] effects. At the molecular level, metformin has been shown to inhibit mTOR1 [57], ERK [58], and raf-ERK signaling [59]. Due to these properties, metformin has been successfully demonstrated to ameliorate liver injury caused by drugs like acetaminophen [60], arsenic trioxide [61], and endotoxins [62] in animal models. A recent study also reported metformin to improve fenestrations on liver sinusoidal endothelial cells, ultimately enhancing their function [63]. These properties make metformin an ideal candidate for ameliorating Bu-mediated liver toxicity. Indeed, we observed metformin to reduce ROS production, ERK(1/2) activation, and apoptosis on LSECs, ultimately improving their functionality as evidenced by FITC-albumin uptake. These results indicate the possibility of repurposing metformin in the HSCT setting as it ameliorates Bu-mediated damage and does not hamper the engraftment potential when transplantations are done.

Interestingly we did not observe such an extent of extramedullary toxicity in the livers of Treo-treated mice, although the dose used was myeloablative. We also observed megakaryocytes in the spleens of Treo-treated mice, which could potentially mediate recovery post myeloablative injury. Surprisingly, megakaryocytes were also seen in metformin pretreated mice that received a myeloablative dose of Bu, indicating a similar recovery mechanism post-metformin treatment.

Despite these encouraging results, it should also be noted that the mice models we used are immunocompetent wild-type Balb/c. Although it closely mimics the HSCT setting, there are limitations like using combinations of conditioning regimen drugs such as fludarabine/Bu or fludarabine/Treo and performing HSCT in a hematological disease model. Nevertheless, we believe this simple model paved the way for identifying molecular mechanisms of Bu-mediated liver damage and possibly reversing the same. These findings could be further tested using a combination of drugs in a disease model.

In conclusion, here, we tried to elucidate the exact mechanisms of toxicities of Bu and Treo on endothelial cells. Although with differential extramedullary toxicities, both Bu and Treo are still used in the HCT clinic for various hematological conditions. In this context, it is imperative to know the molecular mechanism of toxicities to alleviate them in the clinic. In this study, though Bu and Treo were found to induce DNA damage in liver-specific endothelial cells, Bu alone induced oxidative stress-mediated sustained pERK(1/2) activation leading to apoptosis executed by cleaved PARP and caspases. Based on our study, metformin pretreatment with Bu ameliorates off-target toxicities by decreasing oxidative stress mediated sustained activation of pERK(1/2) while probably supporting the recovery of marrow post-transplantation.

## Materials and methods

### Chemicals, reagents, and antibodies

Busulfan (Busilvex) and Treosulfan were purchased from Otsuka Pharmaceuticals and Medac GmBH, respectively. Metformin, N-acetyl cysteine (NAC), rosiglitazone, Hoechst 33342, and albumin-FITC were purchased from Sigma Aldrich (St. Louis, MO, USA). U0126 was purchased from InvivoGen (Toulouse, France). Collagenase type I and DCFDA (2’,7’–dichlorofluorescein diacetate) and DAPI were from ThermoScientific (Waltham, MA, USA). Antibodies used for flow cytometry, immunoblotting, and immunofluorescence are listed in S1 Table.

### Cell culture

Primary Human hepatic sinusoidal endothelial cells (HHSEC; ScienCell, Carlsbad, CA, USA) were cultured on 1% gelatin-coated surfaces in endothelial cell media (ScienCell) supplemented with 5% fetal bovine serum (FBS) (ScienCell), endothelial cell growth supplement (ECGS; ScienCell) and 100 U/mL penicillin, and 100 μg/mL streptomycin (ScienCell). Human umbilical vein endothelial cells (HUVEC; Manassas, VA, USA) were cultured on 1% gelatin-coated surfaces in M199 media (Sigma-Aldrich, St. Louis, MO, USA) supplemented with 50 μg/ml endothelial cell growth supplement (ECGS; Sigma), 10 μg/ml hydrocortisone, 10% FBS and penicillin-streptomycin (Gibco, Carlsbad, CA, USA). SK-HEP1 cells of liver endothelial origin [64, 65] were cultured in Eagle’s Minimum Essential Medium (EMEM; Sigma) supplemented with fetal bovine serum (10%) and 10% FBS and penicillin-streptomycin (Gibco). All cells were cultured in a humidified atmosphere with 5% CO_2_ at 37°C.

GCSF mobilized peripheral blood stem cells obtained from healthy volunteers were enriched for CD34+ cells using EasySep human CD34 positive selection kit (Stemcell Technologies, Vancouver, Canada) and cultured using IMDM medium. All cell culture experiments were done in triplicates with three independent experiments.

### Mice

In-bred Balb/c mice housed in individually ventilated cages at 22–24°C in the laboratory animal facility (CSCR, Vellore) were used. All experiments were performed in 8–10 weeks old male/female mice. Experiments complied with ethical regulations and were approved by the institutional animal ethics committee, CMC, Vellore. All experiments were performed in accordance with relevant guidelines and regulations, and the study was conducted in accordance with Animal Research: Reporting of in vivo Experiments (ARRIVE) guidelines.

### Conditioning regimen drugs evaluation *in vivo*

Balb/c mice were injected with myeloablative doses of either Bu (4 x 25 mg/Kg) or Treo (3 x 1000 mg/kg) intraperitoneally, as previously reported [66, 67]. Groups of animals (n = 5) were euthanized on day+1 & day+7 post-drug administration by CO2 asphyxiation. WBCs, CD31+ bone marrow endothelial cells (BMECs), and CD146+ liver sinusoidal endothelial cells (LSECs) were enumerated by flow cytometry. Liver, spleen, and bone marrow sections were evaluated by histology and immunofluorescence. Untreated Balb/c mice of the same age group were used as controls.

In a separate set of experiments, three groups (n = 5 each) of mice were used to evaluate the pharmacological attenuation of Bu toxicity. Mice were either pre-treated with metformin (200mg/kg) on day-4 and days -3, -2, -1, 0, one hour prior to Bu treatment (metformin + Bu group) or treated with metformin alone on days -4, -3, -2, -1, 0 (metformin alone group). Mice were euthanized on day+1 and day+7 post-treatment by CO2 asphyxiation for further histological evaluation.

For rescue transplant experiments, groups of (n = 5 each) female BALB/c mice were treated with either busulfan or metformin + busulfan at doses as described above. On day+1, these mice were transplanted with 1 x 10^6^ bone marrow cells from male BALB/c donor mice. Eight weeks post-transplantation, peripheral blood from transplanted mice was collected by retroorbital bleeding. Chimerism analysis was performed in sex-mismatched transplants by quantitative real-time PCR evaluation of the Y chromosome-specific gene, Zfy1, as described previously [68].

### Flow cytometry

#### Detection of apoptosis

SK-HEP1, HUVEC, or HHSECs were seeded at a density of 4–6 x 10^4^ cells/well in a 24-well plate. The cells were then treated with varying concentrations of either Bu (200 μM, 300 μM, and 500 μM) or Treo (3 μM, 10 μM, and 30 μM) for 48h [doses decided based on previous publications] [35, 69]. Apoptosis was determined by staining the cells with Annexin V-allophycocyanin (APC) and 7AAD (BD Pharmingen, San Diego, CA) per the manufacturer’s instructions. The cells were acquired using the Accuri C6 cytometer (BD Biosciences, Franklin Lakes, NJ) and analyzed using BD Accuri C6 software (version 1.0.264.21).

#### Detection of oxidative stress

SK-HEP1 cells were treated at indicated concentrations of Bu or Treo for 8 hours. Reactive oxygen species (ROS) levels were determined by incubating the cells with DCFDA for 15 min, followed by acquiring the cells in a Gallios flow cytometer (Beckman Coulter, USA). The data were analyzed using Kaluza software version 2.0 (Beckman Coulter, USA). Inhibition of ROS was analyzed by pre-treating the cells with N-acetyl cysteine (NAC) (1mM) or metformin (2mM or 5mM) for 1 hour prior to Bu treatment.

#### Cell cycle analysis

SK-HEP1 cells were treated with either Bu (300 μM) alone or pre-treated with metformin (2mM) or U0126 (10 μM) before Bu treatment. The cells were fixed and permeabilized using methanol, followed by incubation with DAPI (10 μg/ml). Cell cycle status was determined by acquiring the cells in a Gallios flow cytometer (Beckman Coulter, USA), and data were analyzed using Kaluza software version 2.0 (Beckman Coulter, USA).

### Immunofluorescence

SK-HEP1 cells grown at a density of 60,000 cells in a poly-L-lysine coated glass-bottom dish (SPL life sciences, Korea) were treated with Bu or Treo as described above. The cells were then washed, fixed in 4% paraformaldehyde, permeabilized with 0.5% Triton X-100, followed by blocking with 0.2% bovine serum albumin (BSA). The primary and secondary antibodies used are listed in S1 Table. Nuclei were counterstained with Hoechst 33342. Images were acquired at 60X with oil immersion under Olympus FluoView FV1000 confocal laser scanning microscope.

The formalin-fixed and processed liver tissues from untreated control mice, Bu/Treo-treated mice, or mice that received metformin and Bu were sectioned in poly-L-lysine coated slides. The sections were de-paraffinized and rehydrated in xylene, followed by graded ethanol. Sections were blocked in 1% horse serum and stained with primary antibodies and Alexa fluor conjugated secondary antibodies listed in S1 Table. Slides were washed and mounted with Vectashield antifade mounting medium with DAPI (Vector Laboratories, CA, USA) and imaged using Olympus FluoView FV1000 laser scanning confocal microscope at 60X oil immersion objective.

### Immunoblotting

Drug-treated SK-HEP1 cells were lysed with radioimmunoprecipitation assay (RIPA) buffer containing protease inhibitor mixture (1X) (Roche, IN, USA) and 2mM Phenyl methyl sulfonyl fluoride (Sigma Aldrich). Immunoblotting with equal amounts of proteins (30ug) was carried out as reported previously [70] (antibodies listed in S1 Table).

### Bone marrow CD31+ cells evaluation by flow cytometry

On day+1 and day +7 post-drug administration, mice were euthanized by CO2 asphyxiation. Femur and tibia were collected and flushed with 1ml sterile PBS with EDTA. The flushed marrow from different groups was then subjected to RBC lysis and washed with 1X PBS. WBCs were then counted and stained for CD31+ cells using the FITCanti-mouse CD31 antibody. After incubation, the cells were washed and acquired by flow cytometer (BD FACSCelesta, San Jose, CA, USA) and analyzed using FACSDiva 8.0.1.1.

### Evaluation of liver sinusoidal endothelial cells (LSECs)

Post-drug treatment, the animals were euthanized, liver harvested, and the non-parenchymal fractions were separated, as described previously [71]. Briefly, the liver lobes were washed twice, minced thoroughly, and subjected to 0.1% collagenase I digestion at 37°C. Hepatocytes were removed by low-speed centrifugation at 54g. The remaining non-parenchymal cells in the supernatant were pelleted by centrifuging at 640g. These cells were then resuspended in PBS, stained for PerCPanti-mouse CD146, acquired in BD Celesta flow cytometer, and analyzed using FACSDiva 8.0.1.1. UV-CD146+ cells were considered as LSECs.

### Histopathological analysis of tissues

A part of the bone, liver, spleen, kidney, lung, and heart from treated and control mice was fixed in 10% buffered formalin. The fixed tissues were processed, embedded in paraffin, sectioned, stained with hematoxylin/eosin, and imaged using a Zeiss light microscope with a 10x dry objective lens. A certified pathologist reviewed the slides. In the case of bones, the fixed bones were decalcified in 10% EDTA for up to three days before sectioning and staining.

### Confocal imaging of liver vasculature

Mice treated with Bu, Treo, metformin + Bu, or untreated were injected intravenously with FITC-albumin (1mg/mouse) via the tail vein. Thirty minutes post-administration, the mice were euthanized by CO2 asphyxiation. Liver lobes were harvested and rinsed in sterile PBS. Whole mounts of liver lobes were imaged using Olympus FV1000 laser scanning confocal microscope at 60X oil immersion objective. The images were acquired as z-stacks with uniform thickness in optical sections.

### Statistical analysis

Non-parametric *t*-tests were used where appropriate. All statistical analyses were done using GraphPad Prism-v5 software, and a *p*-value *<* 0.05 was considered statistically significant.

## Supporting information

S1 Fig Treo reduces primary CD34+ cell viability at lower concentrations and endothelial cell viability at higher concentrations. **A.** Primary CD34+ cells were enriched from mobilized peripheral blood samples from healthy donors by magnetic sorting. The cells in culture were treated with Treo at 3uM, 10uM, and 30uM. Forty-eight hours post-treatment, the cells were analyzed for apoptosis by annexin V using flow cytometry. **B.** SK-Hep1 cells were treated with higher concentrations of Treo (100uM, 150uM, and 250uM) and analyzed for apoptosis forty-eight hours post-treatment by flow cytometry. *p<0.05 vs. mock-treated cells.
(TIF)

S2 Fig CD31 expression pattern post-Bu or Treo treatment on endothelial cells. SK-HEP1 cells are negative for CD31 expression at the basal level. **A** and **B.** SK-HEP1 cells were treated with Bu at different concentrations and evaluated for CD31 expression by flow cytometry. **C** and **D.** SK-HEP1 cells were treated with Treo at different concentrations and assessed for CD31 expression by flow cytometry.
(TIF)

S3 Fig Bone marrow sections stained with hematoxylin and eosin post-Bu and Treo treatment. (TIF)

S4 Fig Myeloablative doses of Bu and Treo elevates endothelial cell activation markers *in vivo*. Balb/c mice (n = 5) were injected with Bu alone (25mg/kg/day) for 4 days or pre-treated with metformin (200 mg/kg) on day-4 and days -3, -2, -1, 0 one hour before Bu treatment (metformin+Bu group). Another group was treated with Treo (1000mg/kg/day) for 3 days. Untreated mice were used as controls. On day+1 post-drug treatment, the mice were euthanized, and liver lobes were harvested. Total RNA from the liver tissues were extracted and evaluated for endothelial-specific markers’ gene expression by quantitative real-time PCR.
(TIF)

S5 Fig Quantitative assessment of mitochondrial membrane potential on SK-Hep1 cells treated with busulfan and treosulfan. The quantitative measure of mitochondrial membrane potential was analyzed using the ratio of JC-1 dimer (Red) by JC-1 monomer (Green).
(TIF)

S1 Table Materials. (DOCX)

S1 Raw images (PDF)
