# Supplementary figures and images for: Metformin pretreatment ameliorates busulfan-induced liver endothelial toxicity during haematopoietic stem cell transplantation
Source: PLoS One. 2023 Oct 26;18(10):e0293311. doi: 10.1371/journal.pone.0293311 (PMC10602364; doi:10.1371/journal.pone.0293311)

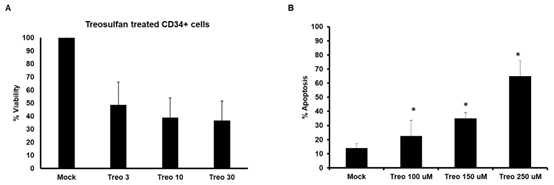

Supplement: S1 Fig — A. Primary CD34+ cells were enriched from mobilized peripheral blood samples from healthy donors by magnetic sorting. The cells in culture were treated with Treo at 3uM, 10uM, and 30uM. Forty-eight hours post-treatment, the cells were analyzed for apoptosis by annexin V using flow cytometry. B. SK-Hep1 cells were treated with higher concentrations of Treo (100uM, 150uM, and 250uM) and analyzed for apoptosis forty-eight hours post-treatment by flow cytometry. *p<0.05 vs. mock-treated cells. (TIF) [file pone.0293311.s001.tif]

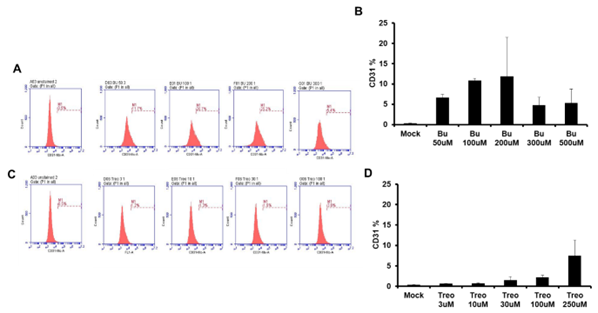

Supplement: S2 Fig — SK-HEP1 cells are negative for CD31 expression at the basal level. A and B. SK-HEP1 cells were treated with Bu at different concentrations and evaluated for CD31 expression by flow cytometry. C and D. SK-HEP1 cells were treated with Treo at different concentrations and assessed for CD31 expression by flow cytometry. (TIF) [file pone.0293311.s002.tif]

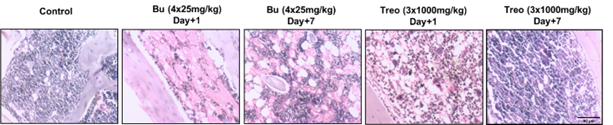

Supplement: S3 Fig — (TIF) [file pone.0293311.s003.tif]

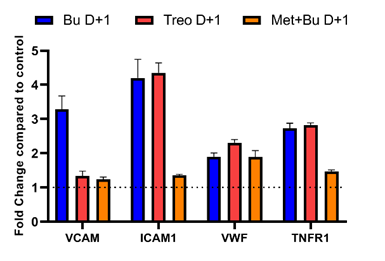

Supplement: S4 Fig — Balb/c mice (n = 5) were injected with Bu alone (25mg/kg/day) for 4 days or pre-treated with metformin (200 mg/kg) on day-4 and days -3, -2, -1, 0 one hour before Bu treatment (metformin+Bu group). Another group was treated with Treo (1000mg/kg/day) for 3 days. Untreated mice were used as controls. On day+1 post-drug treatment, the mice were euthanized, and liver lobes were harvested. Total RNA from the liver tissues were extracted and evaluated for endothelial-specific markers’ gene expression by quantitative real-time PCR. (TIF) [file pone.0293311.s004.tif]

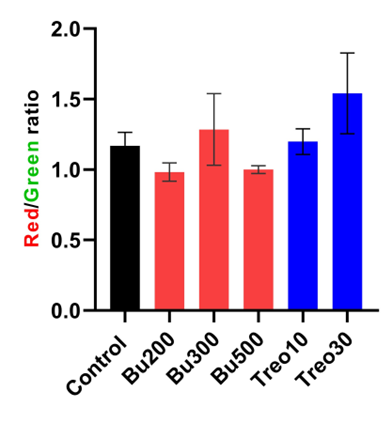

Supplement: S5 Fig — The quantitative measure of mitochondrial membrane potential was analyzed using the ratio of JC-1 dimer (Red) by JC-1 monomer (Green). (TIF) [file pone.0293311.s005.tif]
