# Supplementary material for: Metformin pretreatment ameliorates busulfan-induced liver endothelial toxicity during haematopoietic stem cell transplantation
Source: PLoS One. 2023 Oct 26;18(10):e0293311. doi: 10.1371/journal.pone.0293311 (PMC10602364; doi:10.1371/journal.pone.0293311)
Supplement: S1 Table — (DOCX) [file pone.0293311.s006.docx]

**Supplementary information**

**Table S1**

**MATERIALS**

| **S.No** | **Antibodies** | **Supplier** | **Catalog No.** | **Dilution** |
| --- | --- | --- | --- | --- |
| 1 | Rabbit Anti-Phospho-γH2A.X-S139 | ABclonal | AP0099 | 1:50 |
| 2 | Rabbit Anti-CD31 | ABCAM | ab28364 | 1:50 |
| 3 | Rabbit Anti-CD54/ICAM1 | Cell Signaling | 4915 | 1:1000 |
| 4 | Rabbit Anti-Phospho-p44/42 MAPK (Erk1/2) (Thr202/Tyr204) (D13.14.4E) | Cell Signaling | 8544 | 1:1000 |
| 5 | Rabbit Anti-cleaved caspase 3 (Asp175) | Cell Signaling | 9661 | 1:1000 |
| 6 | Rabbit Anti-Cleaved PARP (Asp214) (D64E10) | Cell Signaling | 5625 | 1:1000 |
| 7 | Rabbit Anti-GAPDH (14C10) | Cell Signaling | 2118 | 1:2500 |
| 8 | Rat Anti-mouse CD146 -PerCP/Cy5.5 | Biolegend | 134709 | 1:10 |
| 9 | Rat Anti-mouse CD31 - FITC | Biolegend | 102405 | 1:10 |
| 10 | Mouse Anti-Hepatic Sinusoidal Endothelial Cells Antibody (SE-1) | NOVUS BIOLOGICALS | NB110-68095 | 1:100 |
|  | **Commercial assay kits** |  |  |  |
| 24 | EasySep Human whole Blood/buffy coat CD34 Positive Selection Kit | Stem Cell Technologies | 18076A |  |
| 26 | High-capacity cDNA reverse transcription kit | Applied Biosystems, Thermo Fisher Scientific, | 4368814 |  |
| 27 | Human sICAM-1 quantikine ELISA kit | R and D systems | DCD540 |  |
|  |  |  |  |  |
|  | **Experimental models: Cell lines** |  |  |  |
|  | **Cells** |  |  |  |
| 28 | Human Hepatic Sinusoidal Endothelial Cells (HHSEC) | SCIENCELL | 5000 |  |
| 29 | SK-HEP1 | ATCC | ATCC-HTB-52 |  |
| 30 | Primary Umbilical Vein Endothelial Cells; Normal, Human (HUVEC) | ATCC | PCS-100-010 |  |
|  | **Experimental models: Organisms/strains** |  |  |  |
| 31 | Mouse: Balb/c | The Jackson Laboratory | JAX:000651 |  |
|  | **Software and algorithms** |  |  |  |
| 32 | Kaluza 2.1 | Beckman Coulter | https://www.mybeckman.in/flow-cytometry/software/kaluza |  |
| 34 | GraphPad Prism 6 | GraphPad | <https://www.graphpad.com/scientificsoftware/prism/> |  |
| 35 | ImageJ |  | https://imagej.nih.gov/ij/download.html |  |
