# Supplementary material for: Metformin pretreatment ameliorates busulfan-induced liver endothelial toxicity during haematopoietic stem cell transplantation
Source: PLoS One. 2023 Oct 26;18(10):e0293311. doi: 10.1371/journal.pone.0293311 (PMC10602364; doi:10.1371/journal.pone.0293311)
Supplement: S1 Raw images — (PDF) [file pone.0293311.s007.pdf]

Full unedited blot for Figure 1 panel E

HHSEC + Busulfan

ICAM1

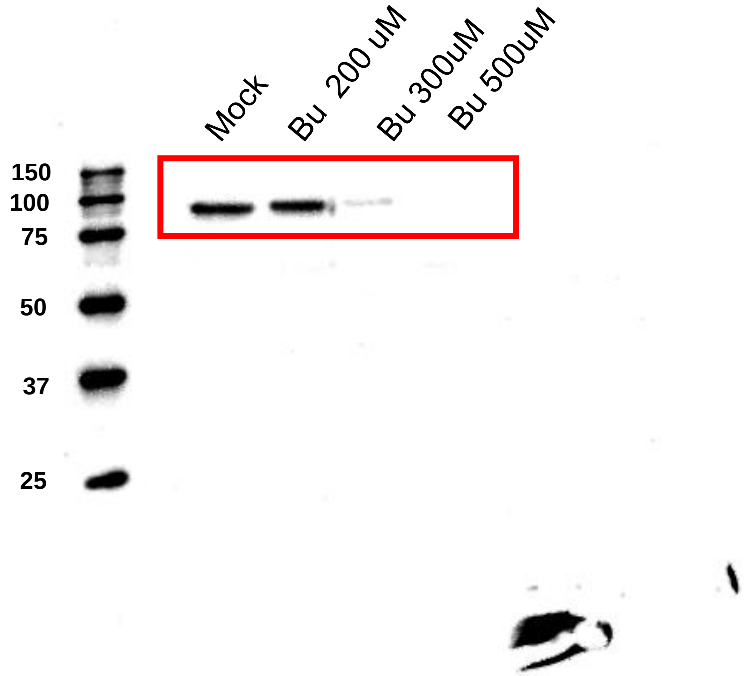

Antibody used: Rabbit anti CD54/ICAM1 (Cell Signaling)

HHSEC + Busulfan

Gapdh

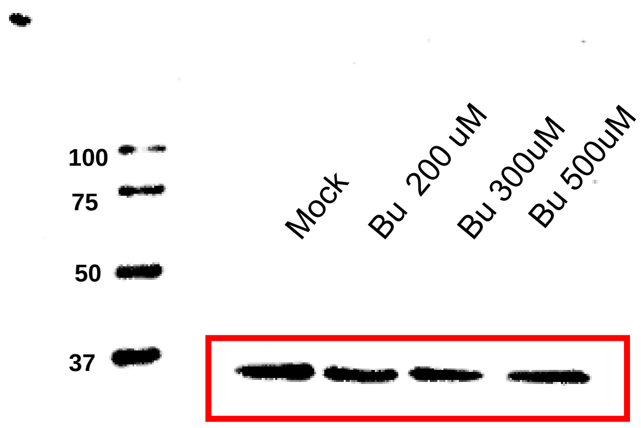

Rabbit anti-Gapdh (Cell Signaling)

Full unedited blot for Figure 1 panel F

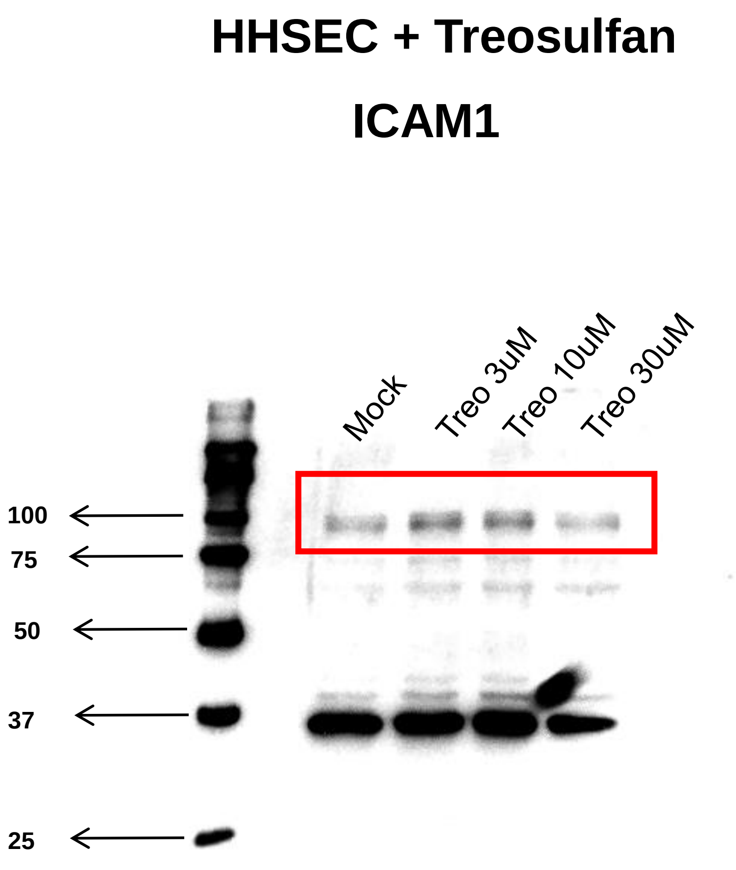

Antibody used: Rabbit anti CD54/ICAM1 (Cell Signaling)

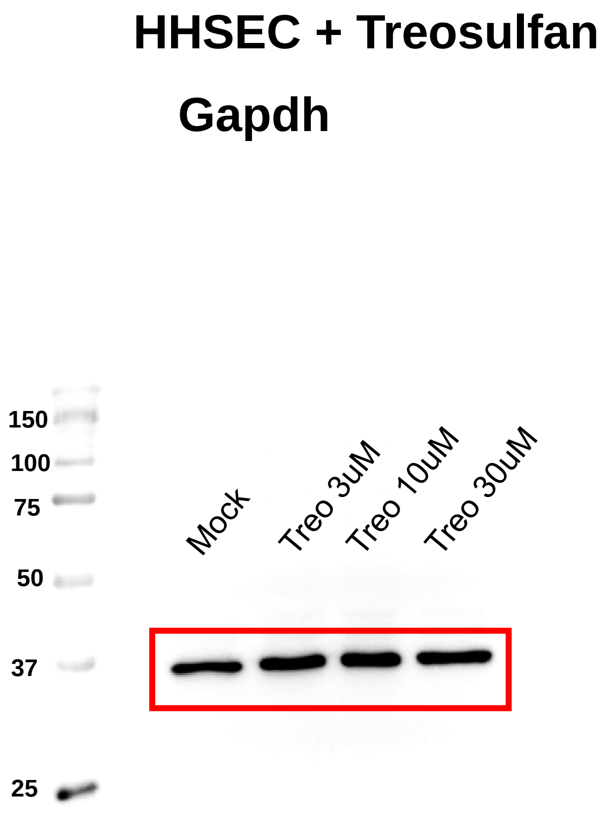

Rabbit anti-Gapdh (Cell Signaling)

Full unedited blot for Figure 1 panel H

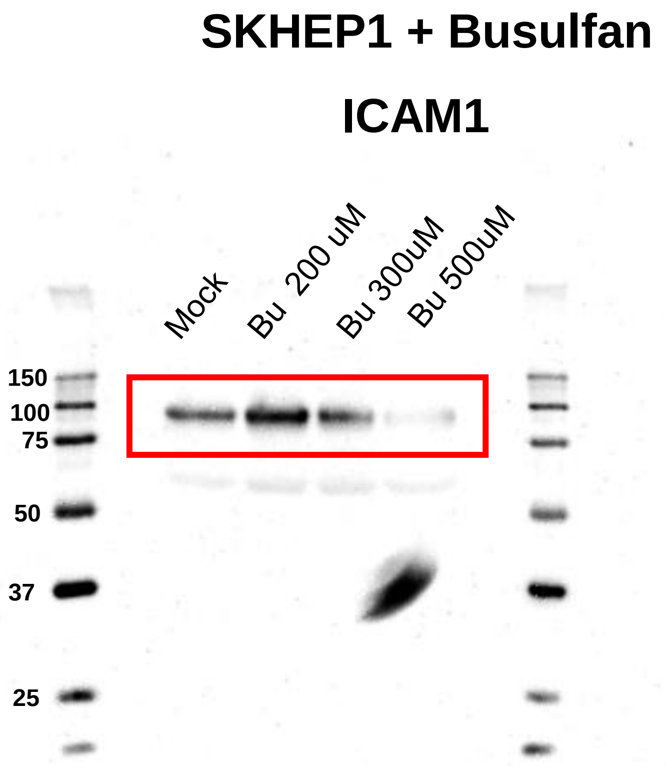

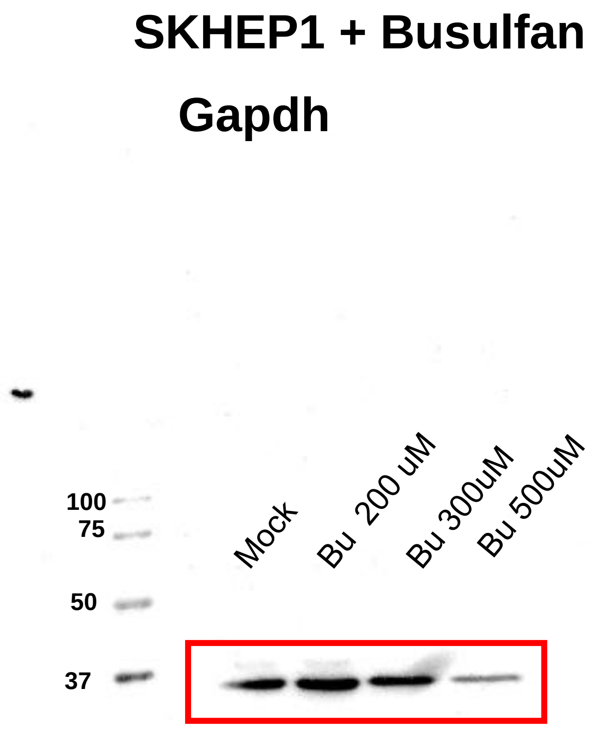

Antibody used: Rabbit anti CD54/ICAM1 (Cell Signaling)

Rabbit anti-Gapdh (Cell Signaling)

Full unedited blot for Figure 1 panel I

SKHEP1 + Treosulfan

ICAM1

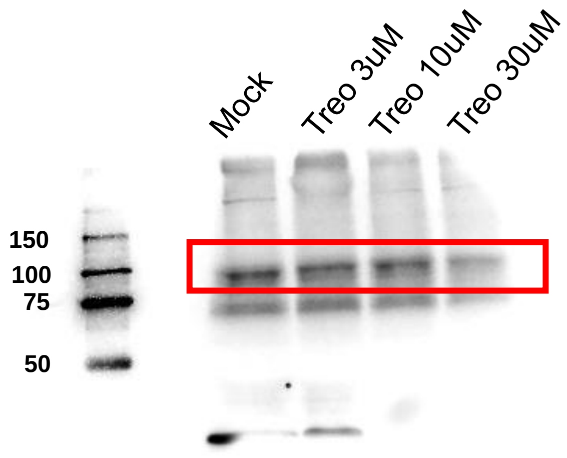

Antibody used: Rabbit anti CD54/ICAM1 (Cell Signaling)

SKHEP1 + Treosulfan

Gapdh

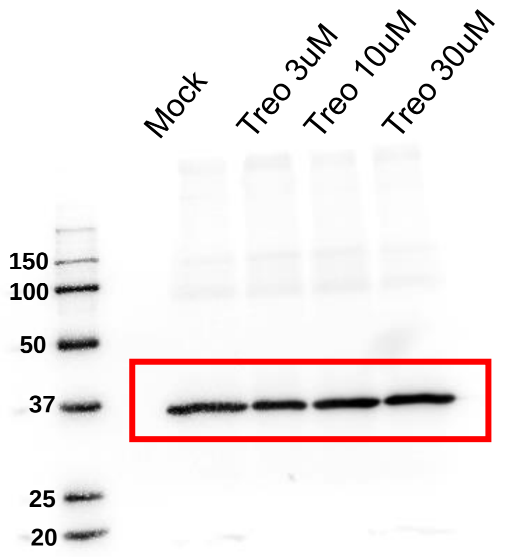

Rabbit anti-Gapdh (Cell Signaling)

Full unedited blot for Figure 2 panel c

SKHEP1 + Busulfan

Cleaved PARP expression

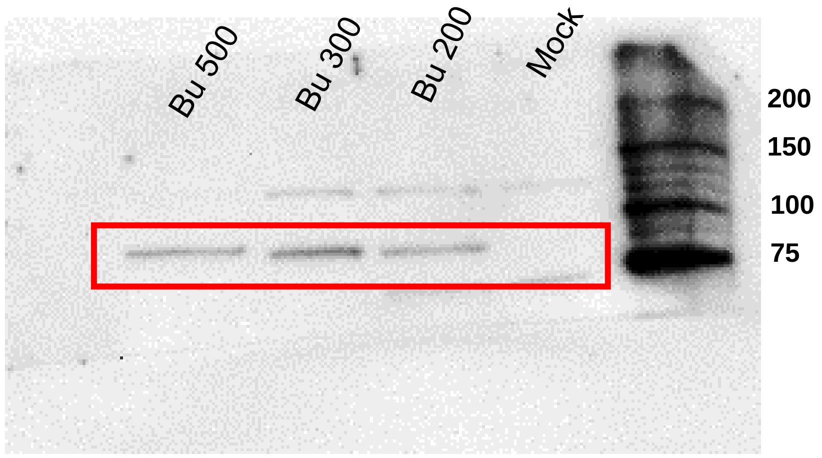

Antibody used: Anti-Cleaved PARP (Asp214) (D64E10) (Cell Signaling)

Cleaved Caspase 3

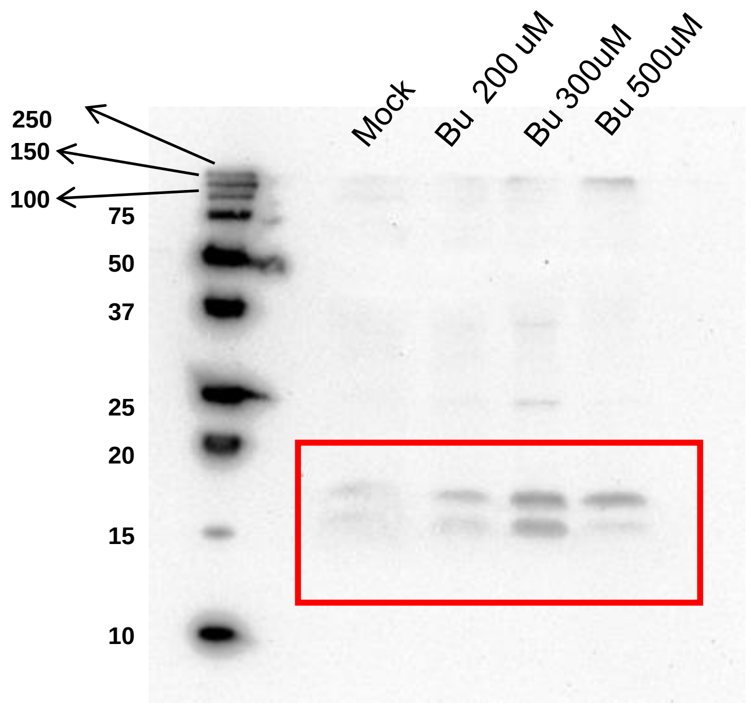

Antibody used: Rabbit Anti-cleaved caspase 3 (Asp175) (Cell Signaling)

Full unedited blot for Figure 2 panel c

SKHEP1 + Busulfan

Phospho-p44/42 MAPK (Erk1/2) (Thr202/Tyr204)

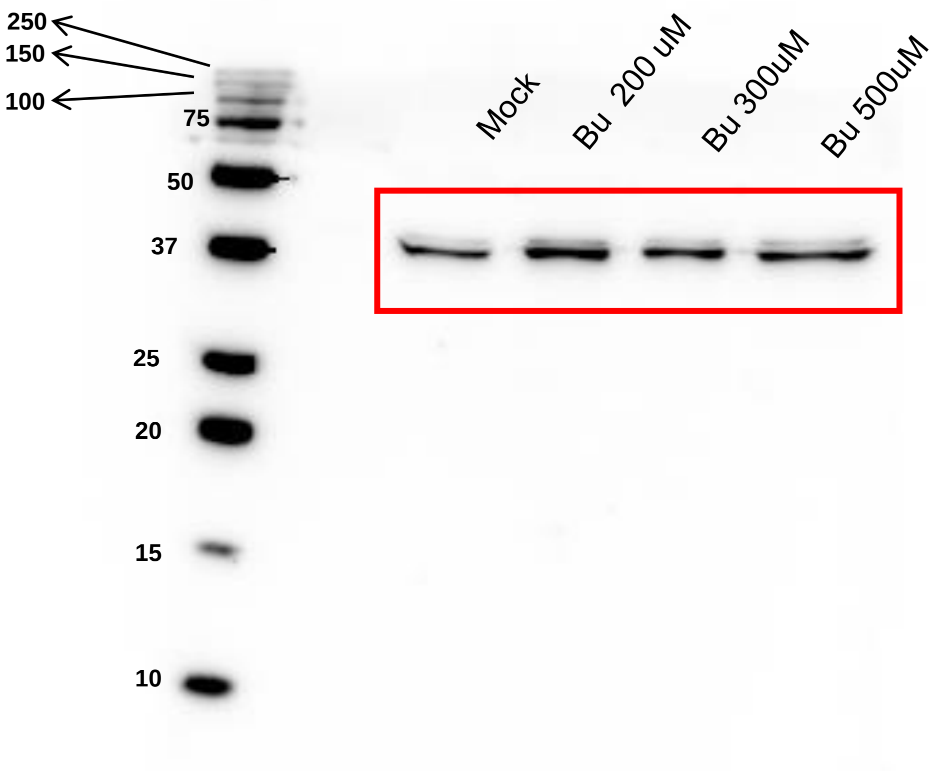

Gapdh

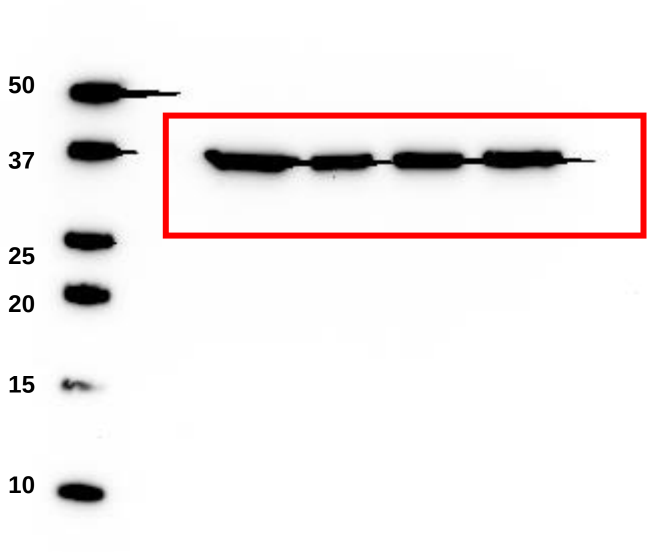

Antibody used: Rabbit Anti-Phospho-p44/42 MAPK (Erk1/2) (Thr202/Tyr204) (D13.14.4E)

Rabbit anti-Gapdh (Cell Signaling)

Full unedited blot for Figure 2 panel D

SKHEP1 + Treosulfan

Cleaved PARP expression

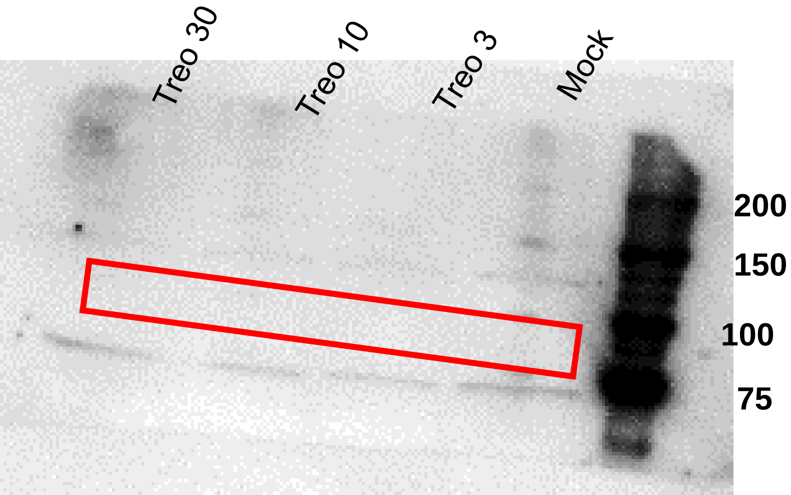

Antibody used: Anti-Cleaved PARP (Asp214) (D64E10) (Cell Signaling)

SKHEP1 + Treosulfan

Cleaved Caspase 3

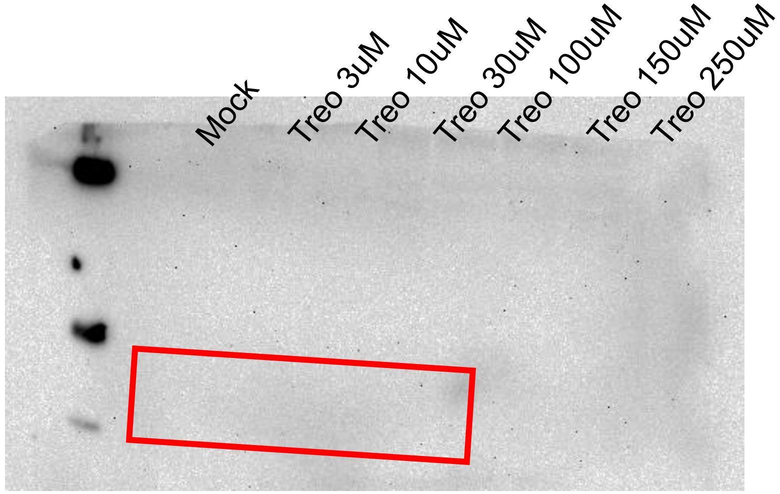

Antibody used: Rabbit Anti-cleaved caspase 3 (Asp175) (Cell Signaling)

Full unedited blot for Figure 2 panel D

Phospho-p44/42 MAPK
(Erk1/2) (Thr202/Tyr204)

<u>SK-Hep1 + Treosulfan</u>

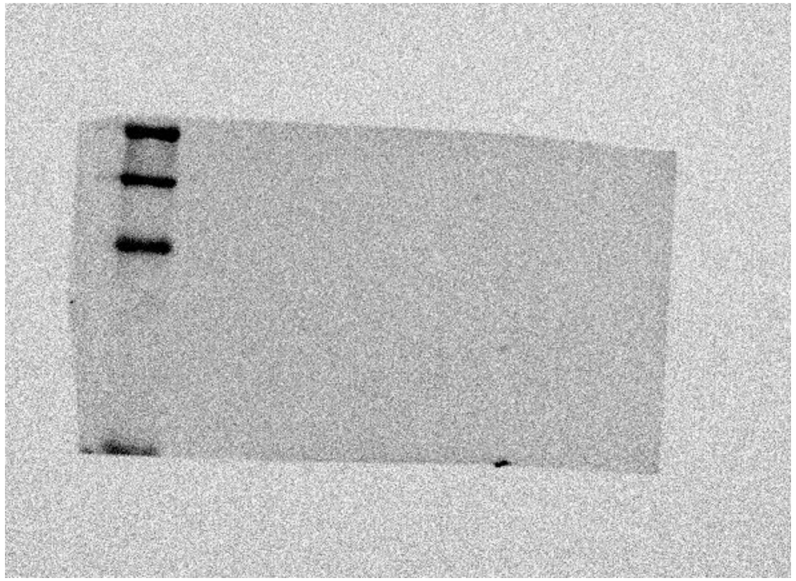

Molecular marker used was not chemiluminescent. Hence a separate image of the same blot in white light is given

<u>SK-Hep1 + Treosulfan</u>

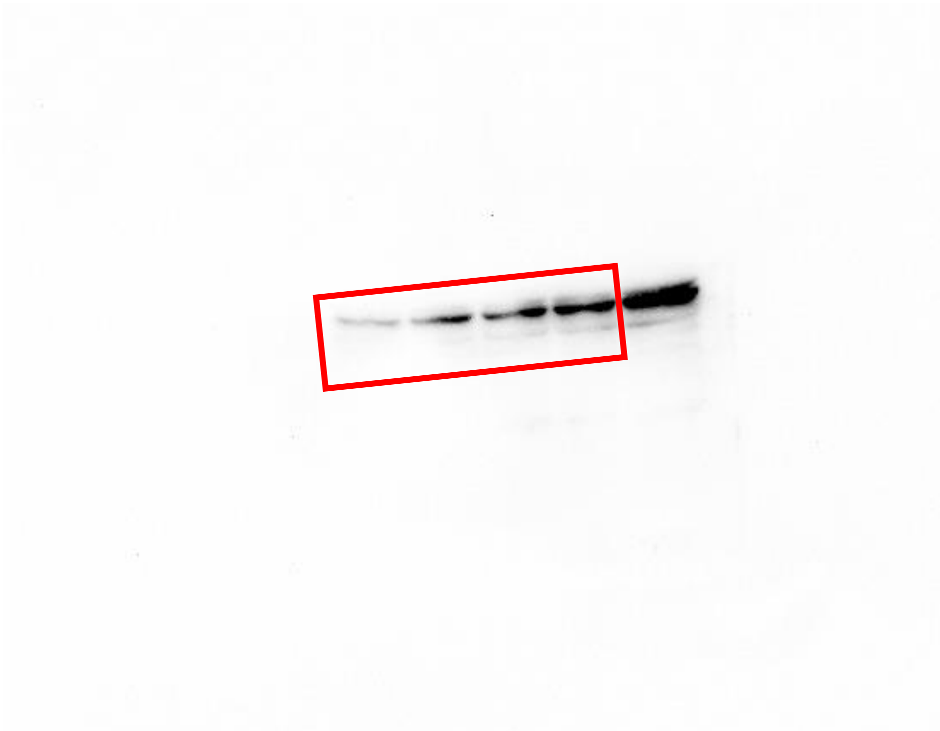

Antibody used: Rabbit Anti-Phospho-p44/42 MAPK (Erk1/2) (Thr202/Tyr204) (D13.14.4E)

Full unedited blot for Figure 2 panel D

SK-Hep1 + Treosulfan

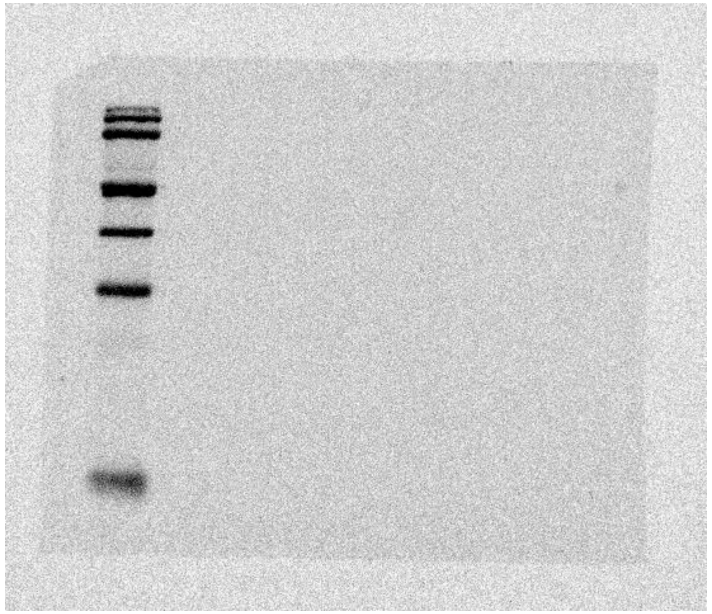

Molecular marker used was not chemiluminescent.
Hence a separate image of the same blot in white light
is given

Gapdh

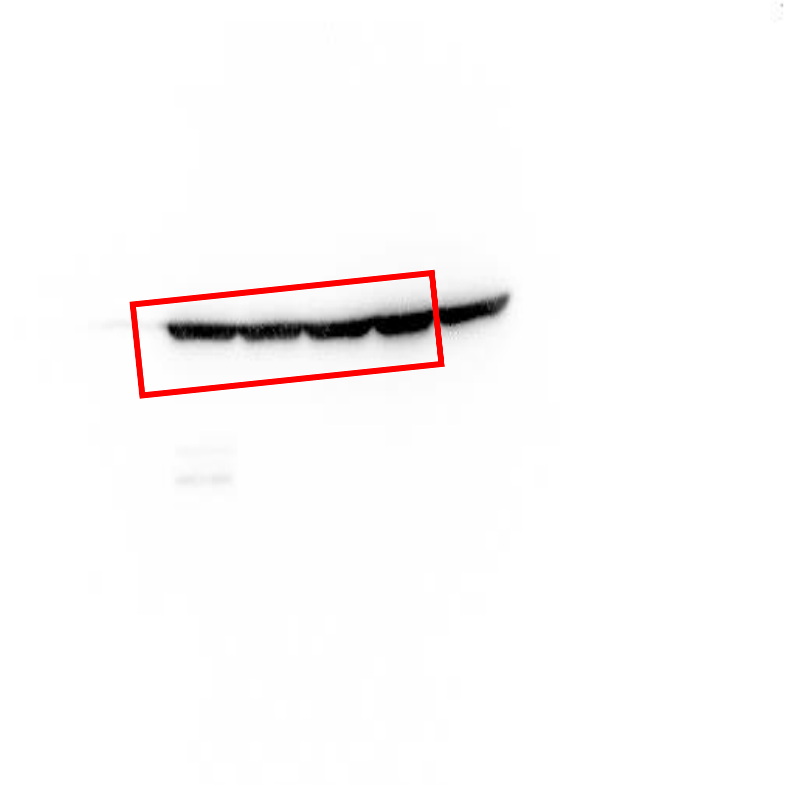

Rabbit anti-Gapdh (Cell Signaling)

Full unedited blot for Figure 3 panel C

Cleaved PARP

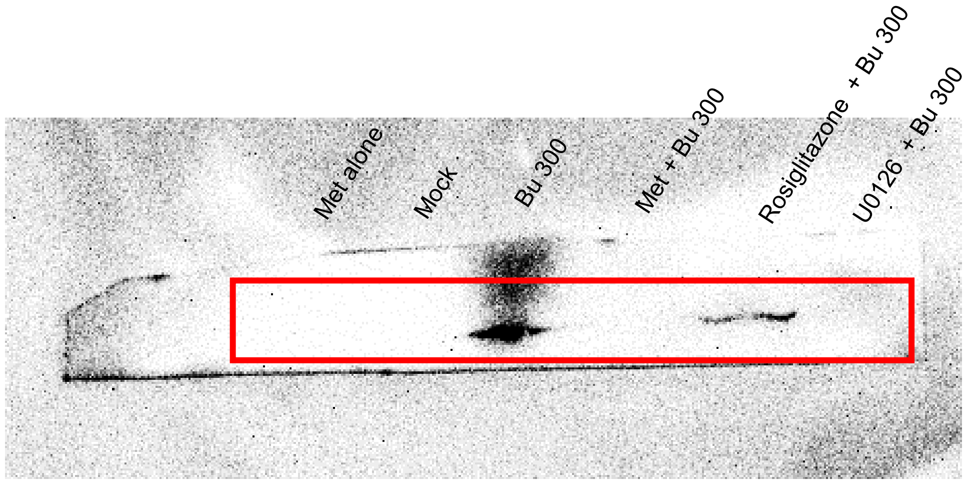

Anti-Cleaved PARP (Asp214) (D64E10) (Cell Signaling)

Cleaved Caspase 3 (18 kDa)

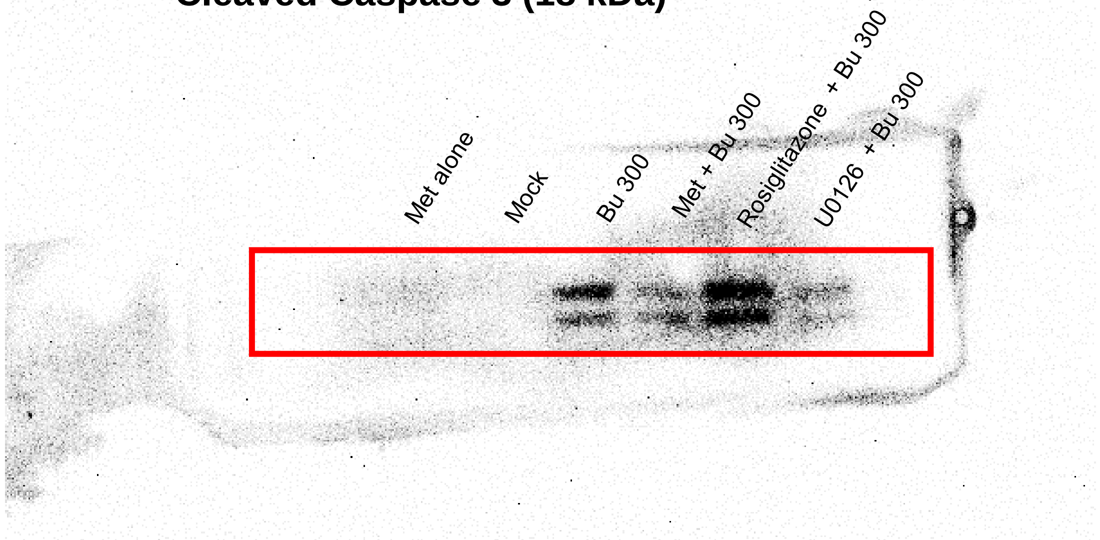

Rabbit Anti-cleaved caspase 3 (Asp175) (Cell Signaling)

GAPDH

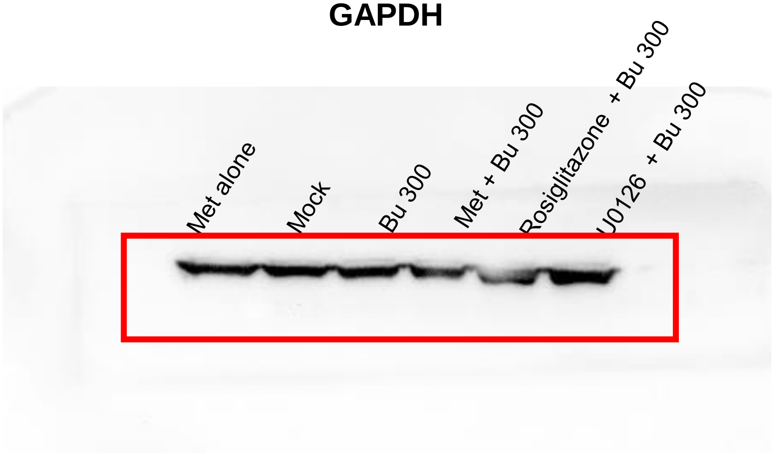

Rabbit anti-Gapdh (Cell Signaling)

Full unedited blot for Figure 3 panel D

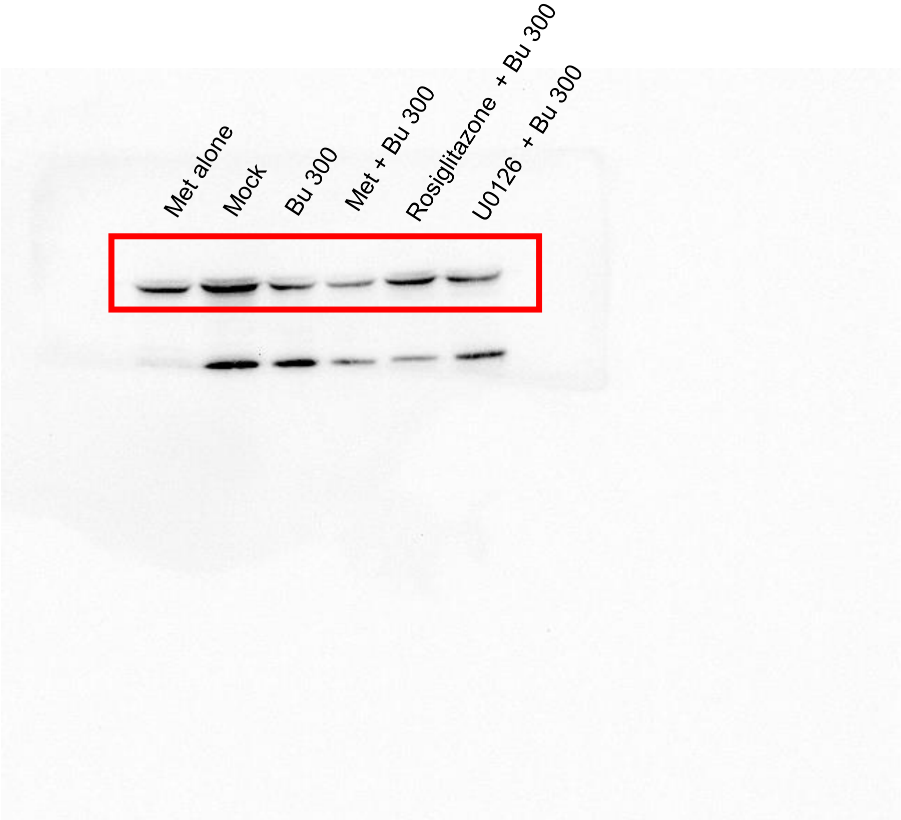

Rabbit Anti-Phospho-p44/42 MAPK (Erk1/2) (Thr202/Tyr204) (D13.14.4E) (Cell signaling)

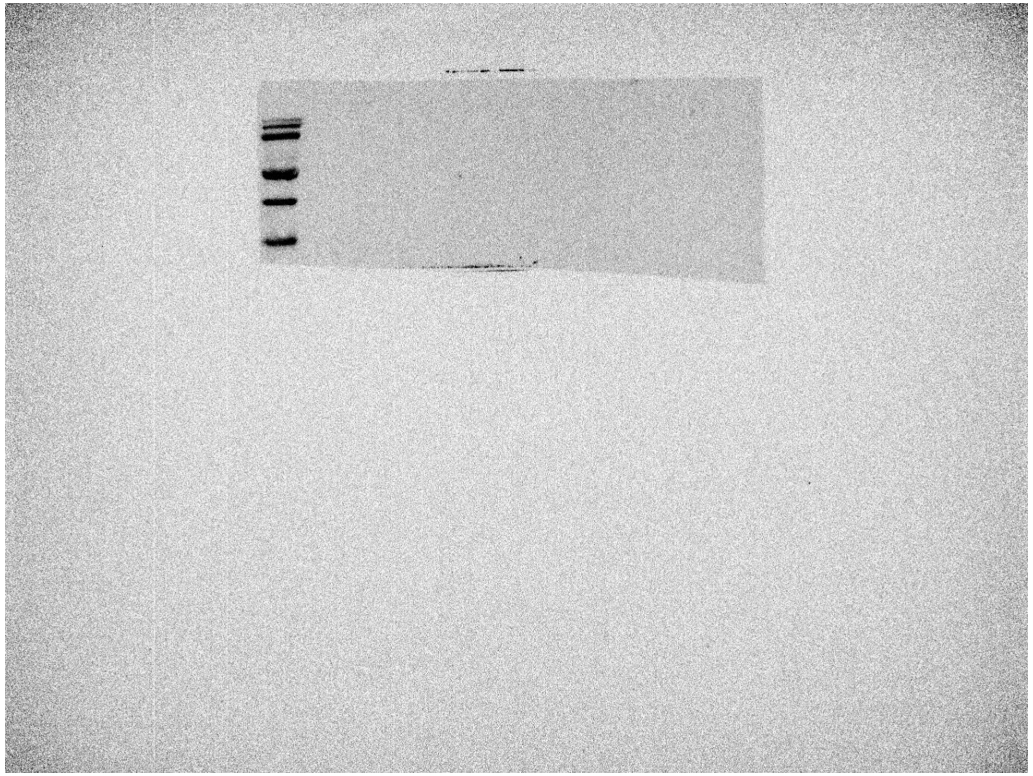

MW marker was not chemiluminescent. Image taken in white light

Full unedited blot for Figure 3 panel D

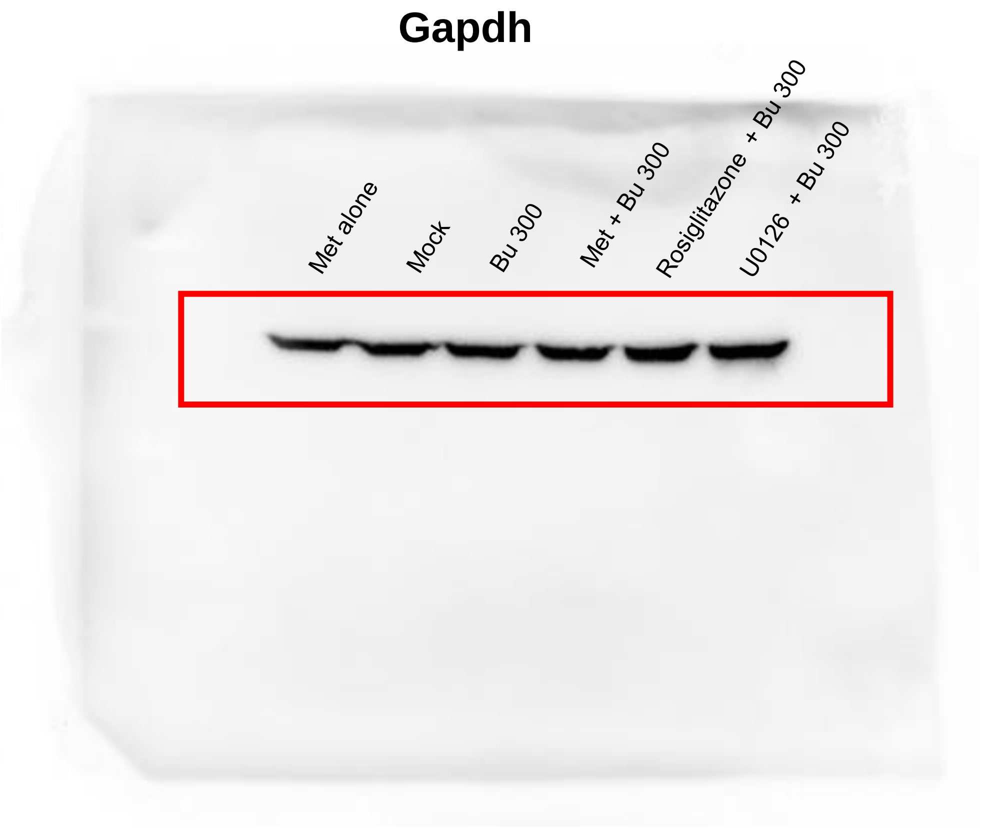

Rabbit anti-Gapdh (Cell Signaling)

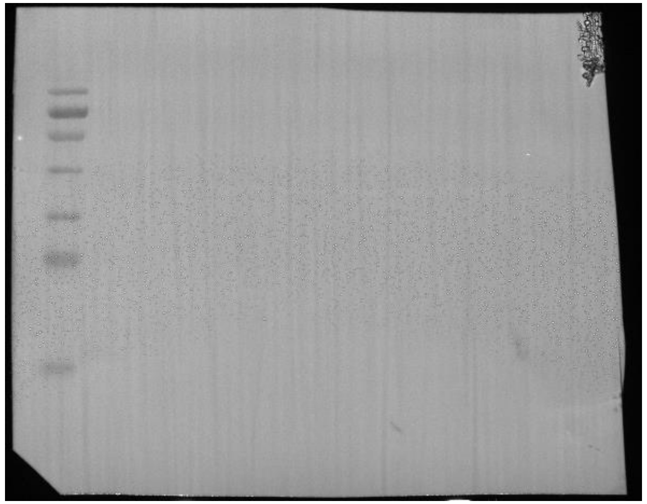

MW marker was not chemiluminescent. Image taken in white light
